# Solubility and Antioxidant Potential of a Pyrogallol Derivative for Biodiesel Additive

**DOI:** 10.3390/molecules24132439

**Published:** 2019-07-02

**Authors:** Hery Sutanto, Bambang Heru Susanto, Mohammad Nasikin

**Affiliations:** 1 Department of Chemical Engineering, Engineering Faculty, Universitas Indonesia, Kampus Baru UI, Depok, Jawa Barat 16424, Indonesia; 2 Department of Chemical Engineering, Faculty of Life Sciences and Technology, Swiss German University, The Prominence Tower, Jalan Jalur Sutera Barat, Alam Sutera Tangerang Banten 15143, Indonesia

**Keywords:** biodiesel, antioxidant, pyrogallol, oxidation, additive

## Abstract

Biodiesel is a renewable plant-based fuel as an alternative for fossil diesel fuel which has many advantages. However, its high content of unsaturated fatty acid causes an oxidation instability during storage. Numerous additives have been used and developed to overcome this problem such as the application of phenolic compound-based antioxidants. Pyrogallol is reported to be one of the best phenolic antioxidants for biodiesel. Unfortunately, pyrogallol has a low solubility in oil solution. In this research, pyrogallol solubility is increased by preparing a pyrogallol derivative through a reaction between pyrogallol and methyl linoleate in the presence of radical 2,2-diphenyl-1-picrylhydrazyl (DPPH). The spectrophotometric method was used for solubility test. Antioxidant potential was examined using acid value determination during a four-week storage period as well as the Rancimat test to see its performance under accelerated oxidation conditions. The reaction produced a molecule which has a molecular weight of 418 g/mol, representing pyrogallol derivative which has a new C–O covalent bond with methyl linoleate. The result was confirmed by using nuclear magnetic resonance (^1^H-NMR, ^13^C-NMR, and 2D-HMQC) resulting in a molecular structure of methyl (10*E*,12*E*)-9-(2,6-dihydroxyphenoxy)octadeca-10,12-dienoate and its isomer methyl (9*E*,11*E*)-13-(2,6-dihydroxyphenoxy)octadeca-9,11-dienoate with a yield of 12.86% and selectivity of 21.05% on the basis of pyrogallol. Compared to pyrogallol, tert-butylhydroquinone (TBHQ), and gallic acid, the pyrogallol derivative has the highest solubility and acid value stability in palm oil biodiesel. The Rancimat induction time (IP) result of the pyrogallol derivative is higher than the biodiesel and is above the accelerated oxidation test American Society for Testing and Materials (ASTM) D 6751 standard.

## 1. Introduction

**Figure 1:**
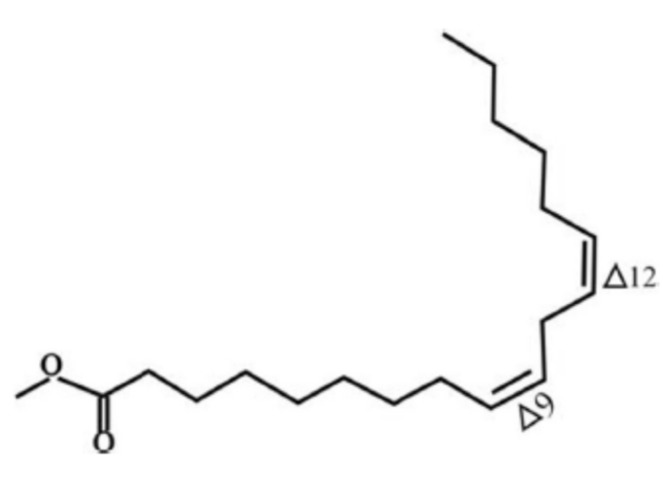
Structure of methyl linoleate.

**Figure 2:**
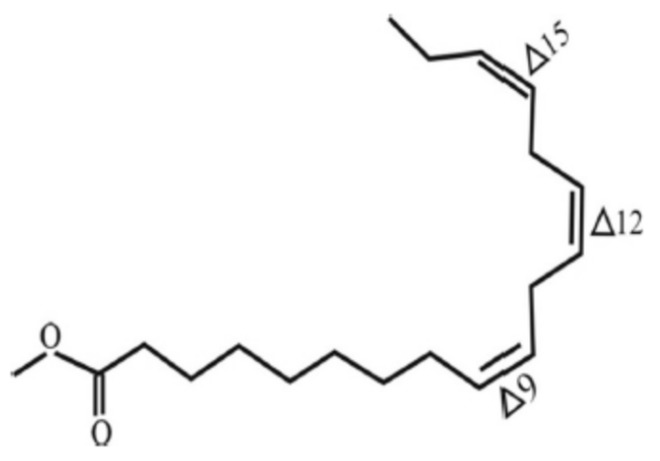
Structure of methyl linolenate.

Biodiesel consists of fatty acid methyl ester (FAME). It is an alternative to conventional diesel which is extensively produced and consumed. It is also in compliance with the governmental regulations in many countries. Despite its numerous advantages, biodiesel has a major disadvantage: instability against oxidation. The oxidation instability of FAME molecules has been a real challenge and the application of additives to increase its stability is of major interest to many researchers [1]. Oxidative instability takes place during the storage period in the presence of pro-oxidant species [2]. As a result of the oxidation, biodiesel molecules could be deteriorated which might leads to quality degradation. The significant effect can be analyzed from the engine performance [3,4]. The natural chemistry of FAME is the main object of this weakness [2]. The methyl esterified long chain of fatty acid is susceptible for oxidation especially in the unsaturated types. Unsaturated fatty acid is fatty acids that have at least one double bond. The degree of oxidation increases as the number of double bonds increase. Figure 1 and Figure 2 are examples of FAME molecules which contain methylene hydrogen at an interrupted position. This methylene hydrogen is the one that will be attacked by radical oxidation because it has the lowest breaking energy due to the resonance potential after the hydrogen abstraction [2,5,6]. This oxidation mostly occurs on polyunsaturated FAME molecules such as methyl linoleate and methyl linolenate which are largely contained in vegetable oils, a raw material source of FAME. One of the products as a result of biodiesel secondary oxidation reaction is the polymerization product which would increase the oil’s molecular mass. The interrupted double bonds resonance into a conjugated isomer and increases the average molecular mass of biodiesel which will increase its viscosity [2].

Various antioxidants have been used to overcome the oxidative instability of biodiesel. Synthetic antioxidants as an additive have been used in different researches. Phenolic antioxidants are widely used as a free radical chain reaction breaker. Many reports show that pyrogallol and tert-butylhydroquinone (TBHQ) are the most effective phenolic antioxidants amongst all phenolic based molecules [6]. From the report by Avase et al. [7], the addition of pyrogallol into biodiesel resulted in better combustion. The superiority of pyrogallol also stated in the report from Karavalakis et al. [8]. Pyrogallol also improved the biodiesel quality in terms of induction period for different biodiesel samples such as rapeseed oil, sunflower oil, used fried oil, and beef tallow [9].

The application of pyrogallol as a biodiesel antioxidant additive has been widely used. However, it has a weakness that the solubility and its stability are low in biodiesel [10] or generally in oil-based samples. Long-chain hydrocarbons of fatty acid methyl ester prevent pyrogallol from completely dispersing in a biodiesel solution because the alkyl group, as a result of the esterification process, decreases the polarity of FAME compared to the original fatty acids. Pyrogallol as a phenolic compound is an organic polar molecule. It has partial negative and positive charges located at the hydroxyl oxygen atoms and the hydroxyl hydrogen atoms respectively. The stable negative ion of pyrogallol property is possessed by the negative charge delocalization by its benzene ring. Moreover, pyrogallol has three hydroxyl groups which contribute to its polarity and will form intermolecular bonds (mostly hydrogen bonding) with other polar molecules, thus it is soluble in polar solvents such as methanol, ethanol, or ethyl acetate [11]. With this low solubility, greater concentration of antioxidant must be added to biodiesel [3], or by surfactant addition to increase the dispersion in biodiesel [12]. Amongst all of the commonly used phenolic antioxidants, pyrogallol has the lowest solubility in biodiesel. The mixing temperature must be increased to 60 °C for 10 minutes in order to dissolve pyrogallol in biodiesel [13]. However, this temperature setting increases the production cost and at the same time risking its antioxidant property. As reported by Santos, N.A., et al [14], increasing temperature leads to deterioration of the phenolic compounds themselves, which will degrade their antioxidant property.

Oil solubility of phenolic compounds has been a problem for more than 80 years. Many research works have been conducted in order to increase the solubility of phenolic compounds in oil-based solutions such as for synthetic resins, dyes, pesticide intermediates, gasoline additives, ultraviolet absorbent, gasoline additives and thermal stabilizers [15]. Wilson, J.C.P [16] invented a method to solve this problem by reacting phenols with alkyl, aryl and unsaturated hydrocarbon molecules using sulphuric acid in order to increase phenolic compounds’ solubility. The research was part of the production of oxidation inhibitor additive for gasoline to prevent gum production. Pyrogallol, catechol, hydroquinone, o-aminophenol, p-amino phenol, p-phenylene diamine, methylamino phenol, and alpha-naphtol were used in the experiment. All of the mentioned compounds are soluble in water and nearly insoluble in gasoline except alpha-naphtol. However, alpha-naphtol itself could not be used since it would change the gasoline color. Substitution of hydrogen atoms in benzene ring with alkyl or aryl group was also reported could increase the additive’s solubility in gasoline. The more alkyl or aryl groups added, the more soluble they were in oil and the more their solubility in water was reduced. The invention from Wilson, J.C.P was instead of using pure alkyl or aryl groups, turpentine and unsaturated hydrocarbons derived from pyrolysis of oil were used as the better nonpolar substitute. As the result, the solubility and storage stability was higher yet the cost lower compared to other simple alkyl or aryl group substitutes. 

Another research was also conducted to increase oil solubility of antioxidants [17]. Several phenolic compounds were modified by the addition of alkyl group to the benzene ring using electrophylic substitution. Some of the synthesized products were also prepared: amyl pyrogallol, butyl methylphenol, propyl gallate, and 5-tert-butylpyrogallol [2]. Other research was done to add alkyl molecules to phenol with branched and linear olefin isomers using acid catalyst in order to increase bio-oil stability [18]. In the research, the liquid-phase alkylation of phenol with linear 1-octene and branched diisobutylene olefins in a batch reactor using homogeneous sulfuric acid and heterogeneous Amberlyst-15 acid catalysts was studied. The research produced ortho-alkyl, meta-alkyl and para-alkyl phenols and also di- and tri-alkylated isomer products. Unfortunately, there is no analysis report about the product’s solubility in oil solutions. Other important research of the coupling reaction between phenolic compound and alkyl molecules in order to increase their solubility and oxidative stability in biodiesel was reported in [19]; the reaction is called synthesis of alkyl phenol using methanesulfonic acid catalyst. This reaction used hydroquinone and catechol to represent phenolic compounds, while the FAME molecules were represented by methyl oleate and ethyl oleate. 

Due to the importance of the needs in biodiesel quality enhancer additive in terms of oxidation stability during storage and considering the result of previous works, this present research aims to obtain a pyrogallol derivative with a high solubility in biodiesel by reacting it with methyl linoleate as a representation of FAME using DPPH reagent without decreasing the antioxidant potential of pyrogallol.

## 2. Result and Discussion

### 2.1. Synthesis Reaction

**Figure 3:**
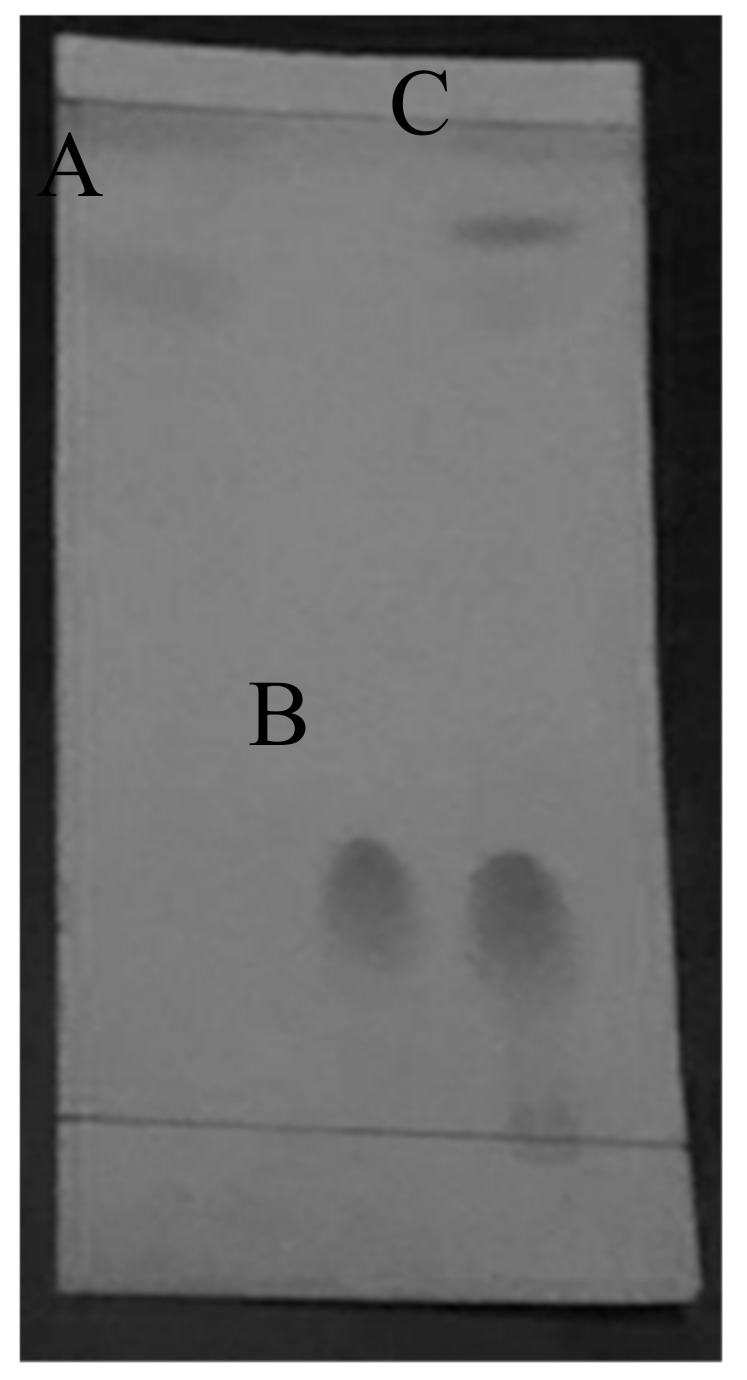
Thin layer chromatography result (**A**): methyl linoleate, (**B**): pyrogallol, (**C**): product.

**Figure 4:**
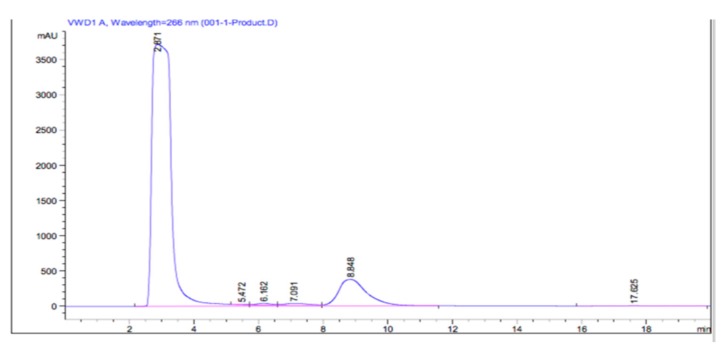
High-performance liquid chromatography (HPLC) chromatogram of pyrogallol derivatives.

Using the thin layer chromatography method, it was confirmed that the reaction product contains a new compound indicated by a new spot appearing on the thin layer chromatography plate at Rf 0.88. This product was compared with the reactant of the products. Meanwhile, methyl linoleate and pyrogallol have different Rf value with the product (Figure 3). This result was suitable given the fact that the product was more non-polar compared to the reactants based on the log P value from ChemDraw Professional 16.0 software. The value of log P or partition coefficient was used as a reference to determine the compound’s relative polarity [20]. The mixture was later subjected to HPLC for the quantitative analysis at its optimum wavelength of 266 as shown in Figure 4. 

**Figure 5:**
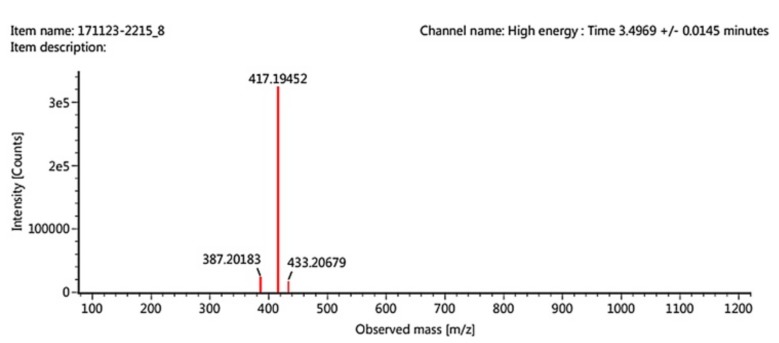
Liquid chromatography tandem mass spectrometry (LCMS-MS) mass spectra.

From the HPLC result, several peaks appeared indicating different components detected in the reaction mixture sample. The pyrogallol derivative product was appeared at the retention time of 8.848 minutes with the percentage by area of 12.86%. Based on the HPLC chromatogram of pyrogallol standard, it is known that the peak at a retention time of 2.871 minutes indicates the presence of unreacted pyrogallol. The peak at 8.848 min fraction was isolated using preparative HPLC and then further analyzed by using LCMS-MS. Figure 5 is the mass spectrum result from the LCMS-MS. The mass to charge (*m*/*z*) value of 417 appeared indicating the molecular mass of the synthesized product. This result confirmed that the condensation reaction between pyrogallol, molecular weight MW = 126 g/mole and methyl linoleate MW = 294 g/mole was successful. The total molecular weight of the new compound was predicted to be 418 g/mole because each molecule lost one hydrogen due to the formation of a new covalent bond. The method used (–H) ion adducts thus *m*/*z* = 417 appears representing 418.

**Figure 6:**
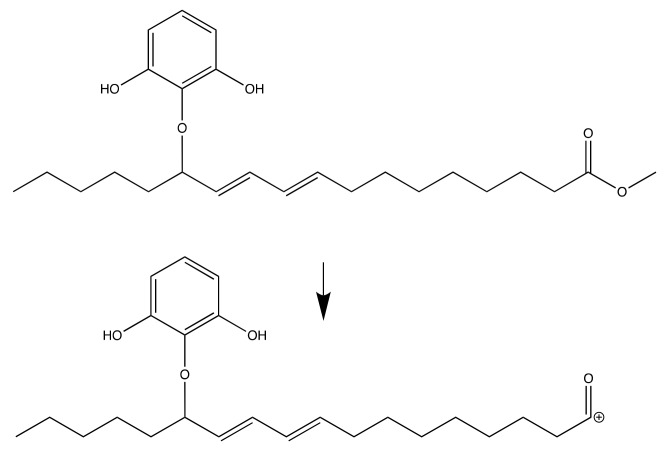
Fragmentation for methyl (9*E*,11*E*)-13-(2,6-dihydroxyphenoxy)octadeca-9,11-dienoate.

**Figure 7:**
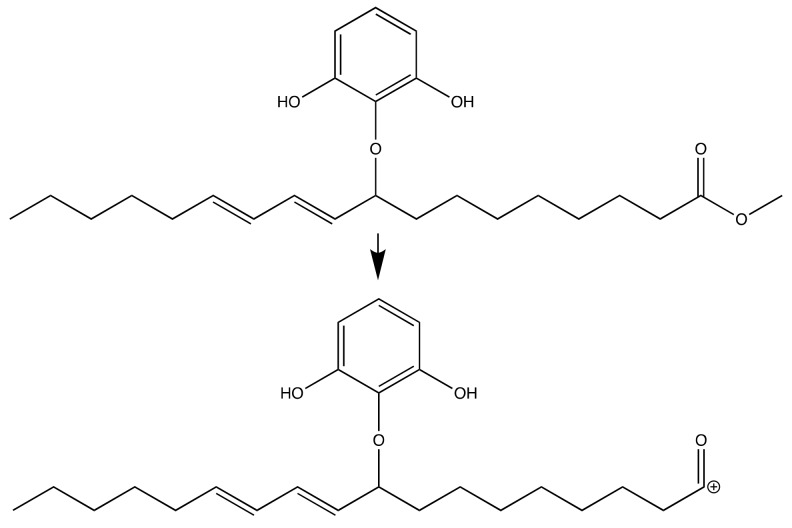
Fragmentation for methyl (10*E*,12*E*)- 9-(2,6-dihydroxyphenoxy)octadeca-10,12-dienoate.

Figure 6 shows the proposed fragmentation of the desired product. These fragmentations fit to the LC-MSMS mass spectra with *m*/*z* = 387. This fragmentation was occurred due to α-cleavage in ester functional group. The similar fragmentation is also proposed for the isomer molecule as can be seen in Figure 7.

**Figure 8:**
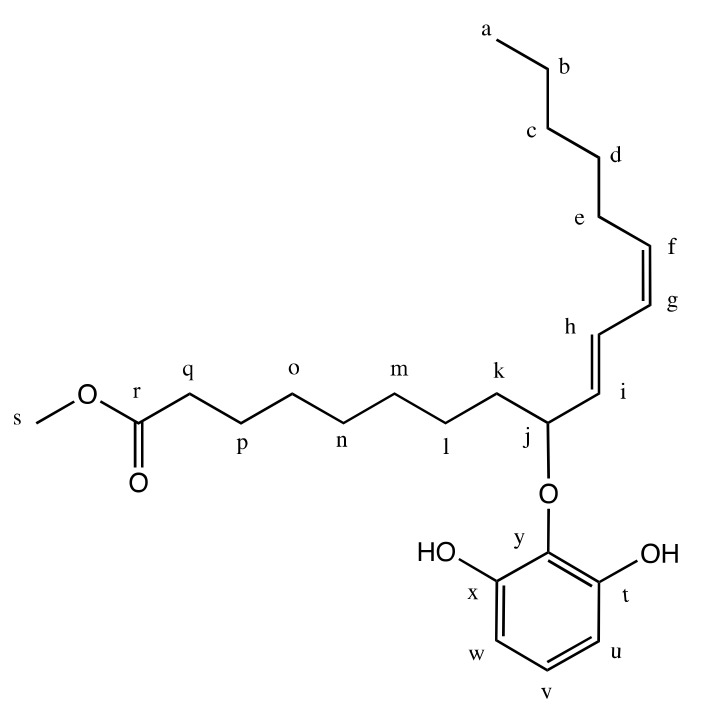
Proposed molecular structure of the synthesis product.

The sample was further subjected to ^1^H-NMR, ^13^C-NMR, and 2D-HMQC analysis to confirm the molecular structure of the synthesized product. The NMR spectrum displayed a series of proton and carbon signals that are match with the predicted molecule as shown in Figure 8.

Using ^1^H-NMR, ^13^C-NMR, and 2D-HMQC (500 MHz, CDCl_3_) which are available in Supplementary Materials as Figure S1, the new compound can be concluded as shown in Figure 8. It is a combination molecule through a formation of a new C–O covalent bond between pyrogallol and methyl linoleate. The new C–O bonding between carbon labelled (j) with the oxygen from pyrogallol is shown in 2D-HMQC (Figure S2) where the quartet signal of ^1^H-NMR at 3.682–3.724 ppm of the (j) proton is correlated with the signal at 58.742 ppm in ^13^C-NMR, the chemical shifts for C-O functional groups. For the attached pyrogallol, its connection with methyl linoleate is through the middle hydroxyl group (carbon y). It is indicated by the symmetrical shape shown by the equivalent signal of carbon (t) and (x), as well as (w) and (u) which show a doublet signal at 6.435 ppm on ^1^H-NMR in correlation with signal 108.175 ppm in ^13^C-NMR as shown by the 2D-HMQC spectrum in Figure S3. Other evidence is the conjugated form of methyl linoleate at carbon (f-g-h-i) shown by multiplet signals at 5.288–5.383 ppm on ^1^H-NMR which has correlations with 4 consecutive ^13^C-NMR signals at 128.092–130.242 ppm (Figure S3). Unfortunately, the singlet signal of hydroxyl protons did not appear which could probably due to the presence of deuterated chloroform solvent, CDCl_3_. The complete set of ^13^C-NMR and ^1^H-NMR signals and chemical shifts is shown in Table 1.

**Figure 9:**
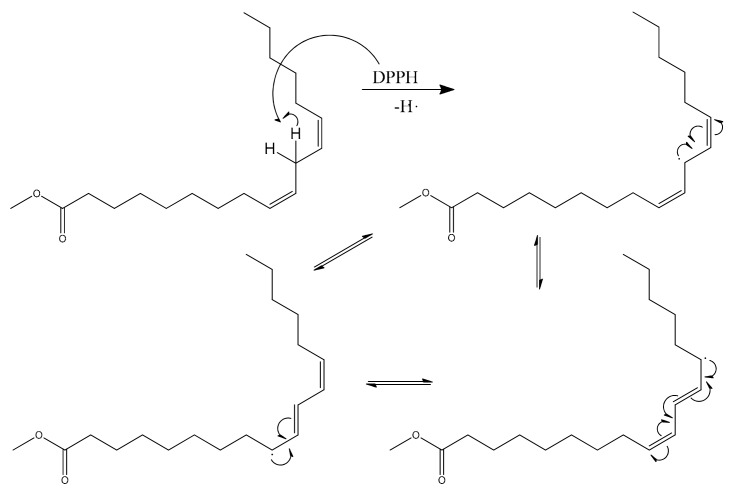
Radical electron delocalization by methyl linolete after hydrogen abstraction by free radical attack.

**Figure 10:**
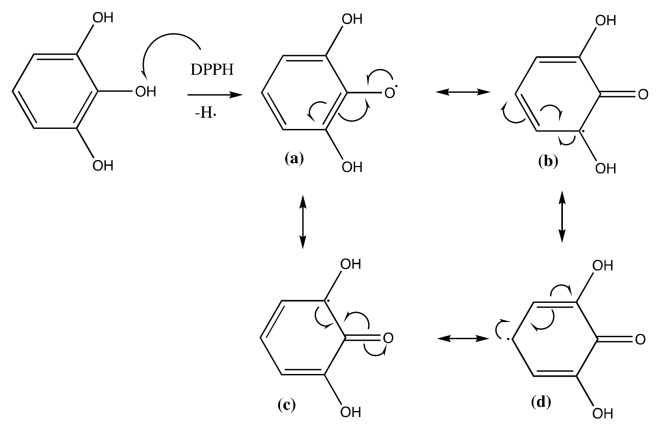
Radical electron delocalization by pyrogallol after hydrogen abstraction by free radical.

**Figure 11:**
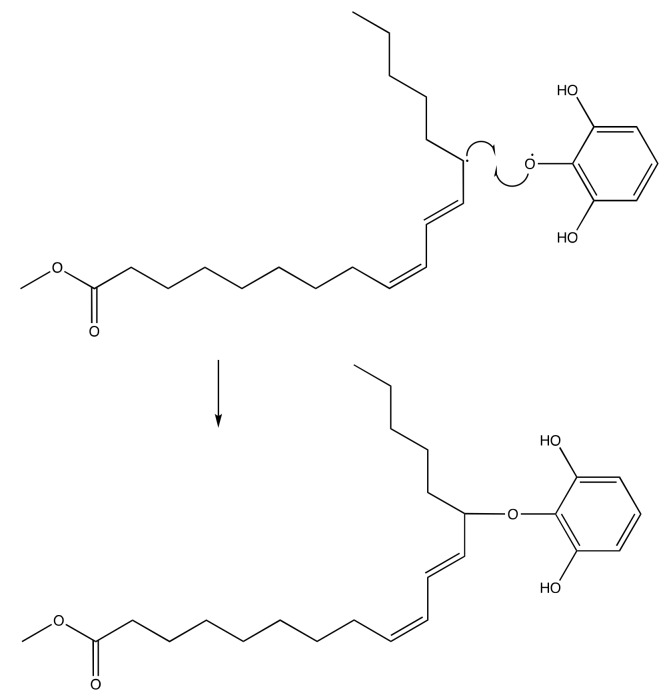
Radical termination mechanism of pyrogallol and methyl linoleate to form methyl (9*E*,11*E*)-13-(2,6-dihydroxyphenoxy)octadeca-9,11-dienoate.

**Figure 12:**
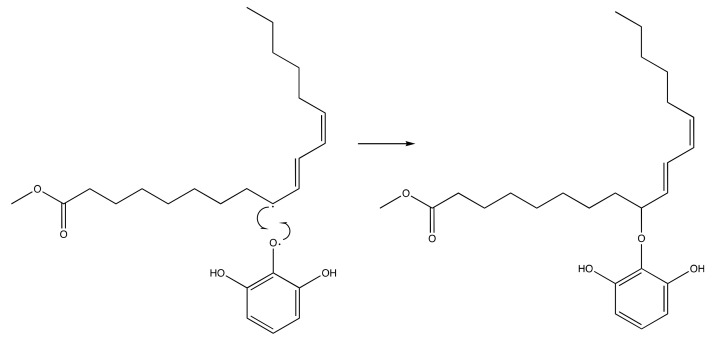
Radical termination mechanism of pyrogallol and methyl linoleate to form methyl (10*E*,12*E*)-9-(2,6-dihydroxyphenoxy)octadeca-10,12-dienoate.

The presence of this new molecule is an evidence that the synthesis reaction was successfully worked. The DPPH radical reacted with both pyrogallol and methyl linoleate, converting them into radical molecules as illustrated in Figure 9 and Figure 10. The radical pyrogallol as shown in Figure 10 delocalizes its singlet electron to different positions to stabilize the molecule before having a termination reaction. Methyl linoleate is a methyl ester form of polyunsaturated fatty acid omega-6 with two double bonds at C-9 and C-12, which means it has a methylene hydrogen at an interrupted position at C-11 (Figure 1). This hydrogen is susceptible to radical attack during the propagation step [6]. After C-11 is radicalized, there will be radical resonance or delocalization to the C-9 and C-13 as shown in Figure 9. The radical pyrogallol and radical methyl linoleate molecules were connected to each other in the termination step forming a new bond with the oxygen atom from pyrogallol resulting molecule methyl (10*E*,12*E*)-9-(2,6-dihydroxyphenoxy)octadeca-10,12-dienoate and its isomer methyl (9*E*,11*E*)-13-(2,6-dihydroxyphenoxy)octadeca-9,11-dienoate molecules as explained by Figure 11. Both molecules have the same chances to be formed due to the two available options of radical methyl linoleate. Both isomer molecules have the same molecular weight of 418 g/mol. The difference is only the new bond location. In Figure 11, the pyrogallol is connected at C13 on the methyl linoleate molecule, while on Figure 12, the pyrogallol is connected at C9 on the methyl linoleate molecule.

### 2.2. Application in Biodiesel

The reaction product, which was expected contains the new molecule, was tested on its solubility and antioxidant activity in biodiesel solution. The antioxidant analysis covers acid number, viscosity, and Rancimat induction period as the common markers for oxidation.

#### 2.2.1. Solubility Test

**Figure 13:**
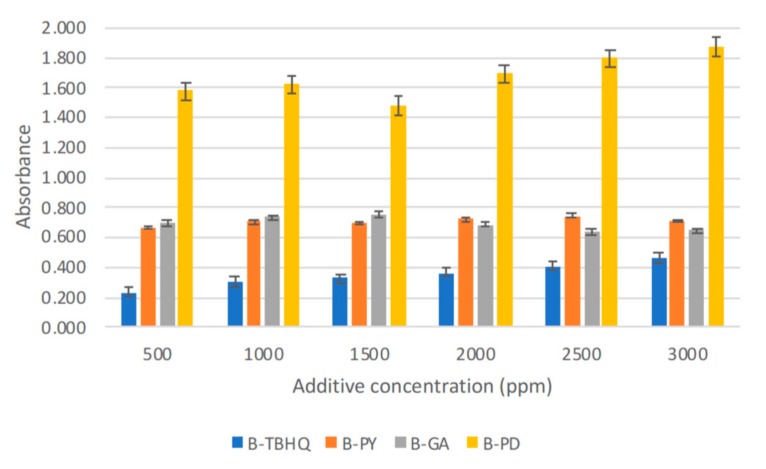
Solubility of different additives in biodiesel.

By using spectrophotometric method, the solubility of four biodiesel-additive mixtures biodiesel-pyrogallol (B-PY), biodiesel-TBHQ (B-TBHQ), biodiesel-gallic acid (B-GA), biodiesel-pyrogallol derivative (B-PD) were measured. As can be seen in Figure 13, starting from the addition of 500 ppm concentration, all additives showed their absorbance values. The general idea of this experiment is that every specific antioxidant concentration was considered soluble if it exhibits a value in terms of absorbance. Therefore, if the addition of higher concentration of antioxidant resulting in an increase of its absorbance, it is considered as still soluble or unsaturated. But when the addition of more antioxidant resulting in a constant value or a decreasing pattern, it is considered as the saturated point, stating that no more antioxidant can be dissolved as a solute. The point before this saturated value is the solubility limit. As can be seen in Figure 13, despite the decreasing absorbance value on the 1500 ppm, B-PD shows another increase of absorbance up to 3000 ppm that is indicated from its absorbance value. The absorbance of PD and TBHQ kept increasing to 3000 ppm while other additives mostly stopped and remained constant, which indicates their solubility had reached the limit.

The new synthesized molecule as a pyrogallol derivative was responsible for the higher solubility of pyrogallol. The methyl linoleate part in the pyrogallol-derivative molecule acts as the molecule tail, increasing the hydrophobic property of pyrogallol in biodiesel, while the phenolic hydroxyl group of the pyrogallol side was proposed to remain unchanged thus it would keep the pyrogallol’s antioxidant property which will be examined in the following experiments. The increased solubility of the pyrogallol derivative mixture is correlated with Log P value of a molecule. A High Log P value indicates a higher fraction would favor organic or nonpolar solvent in an extraction. Log P value analysis results using ChemDraw Professional 16.0 are as follows: pyrogallol (0.87), methyl linoleate (6.23), gallic acid (0.42), TBHQ (2.96), and pyrogallol derivative (6.54). From the log P value, compared to pyrogallol, the pyrogallol derivative is suggested to be more dispersed in the non-polar biodiesel solution.

#### 2.2.2. Acid Number

Acid number is used as an indicator to quantify the number of fatty acids that are present in a biodiesel sample. It expresses the quantity of potassium hydroxide in milligrams that are required to neutralize the free acids present in one gram of substance. The increase of acid number is correlated with the degradation of a sample. It is commonly used as a method to monitor the degradation of biodiesel in terms of organic acid production due to its primary oxidation during storage period [21].

**Figure 14:**
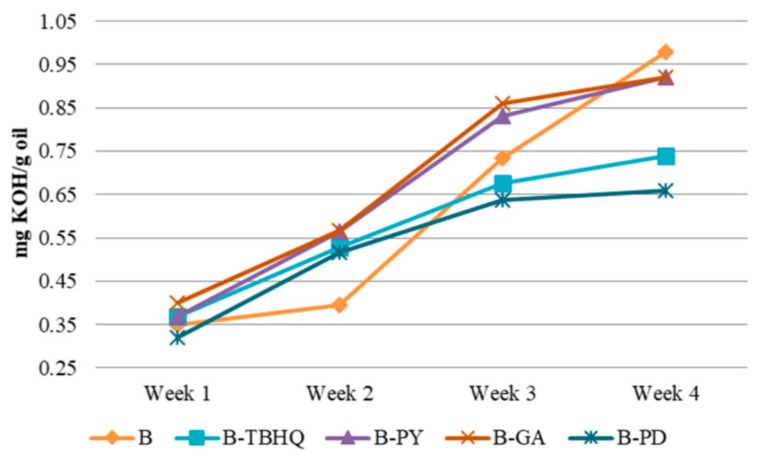
Acid number vs. biodiesel additive.

After the addition of different antioxidant additives, the acid number increased for all samples. Considering the slope values from the linear regression equations of every sample, B has the highest jump (slope = 0.2232) compared to samples with additive. This indicates the oil degradation process occurs most intensively toward biodiesel without additive as can be seen in Figure 14. All additives showed their antioxidant activity by preventing the acid value jump. The lowest acid value in the 4th week was sample B-PD followed by B-TBHQ, B-PY and B-GA with slope values 0.1146, 0.1261, 0.1924, and 0.1850, respectively. The result shows that B-PD has the best activity in maintaining oil quality by preventing the oxidation process indicated by the ability to maintain biodiesels’ acid value. Although all results remain under the United States ASTM D 6751 and European EN 14104 [21], which is < 0.5, it indicates the antioxidant potential of the pyrogallol derivative. As can be seen in Figure 7 and Figure 8, the pyrogallol derivative contains pyrogallol molecule on its hydrophilic end. Although the number of hydroxyl group in the new molecule is reduced compared to the normal pyrogallol that has three hydroxyl substitutes on its benzene ring molecule [22], the remaining two hydroxyl groups on the new molecule keep showing the antioxidant activity of pyrogallol. In the presence of radical species, the hydroxyl hydrogen of phenols will be abstracted by the free radical during the propagation phase [23]. The next propagation step will be stopped by a radical phenol by delocalizing the radical electron into several available positions as shown in Figure 6. This mechanism will protect the phenoxy radical to do further unwanted radical attack or oxidations. In biodiesel, during the exposure of reactive oxygen species, the sample without antioxidant additives will undergo oxidation especially to the unsaturated fatty acids. With the presence of phenolic antioxidant, the biodiesel molecules are protected by the phenolic molecules by scavenging it from the radical attack, meaning that the phenol is the one that will be oxidized and keeps the biodiesel stabilized. With three different hydroxyl groups attached to the benzene ring, pyrogallol has more available acidic hydrogen, which is more susceptible to oxidation compared to other phenolic compounds [24]. Based on Liang et al. as cited in [25], phenols with more -OH groups have better antioxidant activity in comparison with single or two -OH groups. However, when it was compared with PY, PD has better antioxidant property due to the higher solubility of PD in biodiesel solution, thus covering a larger area for protecting the biodiesel molecules against oxidation. Moreover, in this experiment, all sample concentrations were set equally to 1500 ppm or 15mg/100mL biodiesel solution. Therefore, actually PD has a much lower number of phenolics from its pyrogallol side. This expresses how the dispersion of antioxidant is highly important on its activity. Another antioxidant test of the pyrogallol derivative had been reported previously [26] using iodine value, color intensity, and viscosity as the oxidation responses, and the pyrogallol derivative with concentration of 0.1% also showed a positive radical scavenging effect over a four-week storage period.

#### 2.2.3. Viscosity

**Figure 15:**
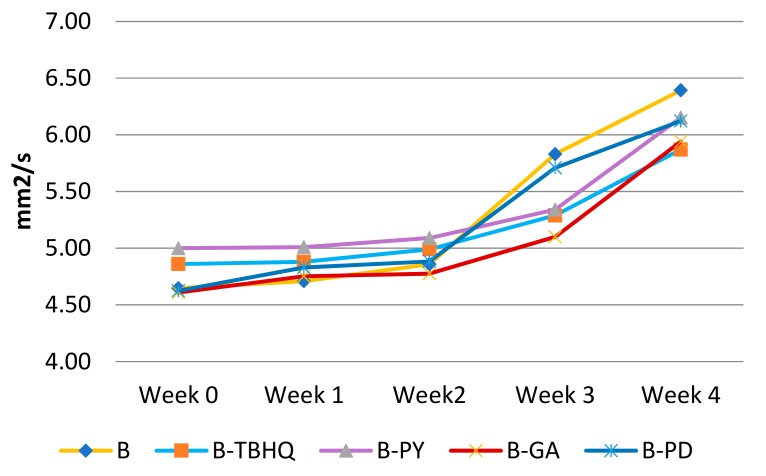
Viscosity vs. biodiesel additives.

Viscosity is a marker of secondary oxidation of biodiesel as a result of polymeric species which are formed by linking fatty acid/FAME chains [21]. The result of the viscosity experiment during four weeks of storage is shown in Figure 15.

After the addition of antioxidants, viscosity of samples increased over the storage period. Comparing the slope jump of the samples, B holds the highest slope of viscosity increase (0.4609), followed by B-PD (0.3873), B-GA (0.3009), B-TBHQ (0.2434) and B-PY (0.2624). Therefore, B-PY is the most effective in slowing down the viscosity increase over time, with B-TBHQ, B-GA and B-PD following. From this result, whilst B-PD does not show the best performance, its antioxidant activity in preventing the formation of polymer product is constantly shown by the fact that it has a lower viscosity jump slope compared to biodiesel without additive. However, although PD was found to be less effective compared to other pure phenolic antioxidants in holding the viscosity value as a marker of secondary oxidation products, its superiority over other antioxidant is proven from the acid number analysis as explained in Section 2.2.2. Understanding that primary oxidation is more likely to happen than secondary oxidation, it is more important to avoid primary oxidation from happening, and PD has been proven to be reliable for this purpose. Therefore, PD can still be considered to have high antioxidant potential.

#### 2.2.4. Oxidation Stability Test (Rancimat Test)

**Figure 16:**
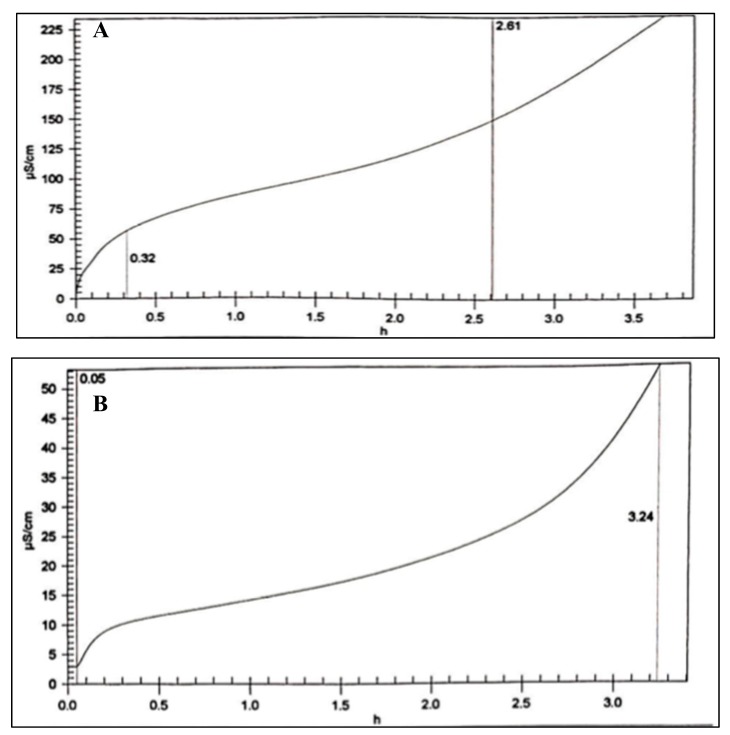
Induction period of (**A**) biodiesel and (**B**) biodiesel combined with pyrogallol derivative using Rancimat method.

In the Rancimat test, two samples were compared: biodiesel without antioxidant; and biodesel with the pyrogallol derivative, PD. It is shown in Figure 16 that PD has higher IP of 3.24 compare to blank biodiesel 2.61. According to the biodiesel standard table in [21], the IP value of PD sample successfully passes the United States ASTM D 6751 standard which is IP > 3. However, based on European EN 14112, it is still under the standard (IP < 6).

## 3. Methodology

The production of pyrogallol derivative is by chemically combining a pyrogallol molecule, which has a relatively high polarity, with one molecule of fatty acid methyl ester representing the nonpolar group. As these two molecules are connected, the new product was expected to have a better solubility in oil-based solutions compared to the original pyrogallol molecule. Based on research by [27], a product result from a reaction of pyrogallol using 2,2-diphenyl-1-picrylhydrazyl (DPPH) produced an oxidative coupling molecule, dimer of pyrogallol. Previously, DPPH has been used mostly for antioxidant assay. It acts as a radical source which will react with phenolic compounds or antioxidants. The fact that the reaction product is a dimer which is an oxidative coupled molecule (polymerization) led to the idea that DPPH can be used as the main reactant for the pyrogallol and methyl linoleate combination. Both pyrogallol and methyl linoleate can react with DPPH [28]. In an oil solution, DPPH method is usually used as an antioxidant assay for unsaturated fatty acids [29]. Methyl linoleate was very reactive with the presence of oxygen when it became radical using DPPH, applying nitrogen gas in the reaction was necessary [30]. The expected product is the result of condensation between two different molecules: pyrogallol and methyl linoleate. The reaction product was expected to be a mixture of dimer or polipyrogallol, dimer or poly-methyl linoleate, and the combination of pyrogallol and methyl linoleate molecules. The reaction product mixture was analyzed using high-performance liquid chromatography, liquid chromatography tandem mass spectrometer (LCMS-MS), and nuclear magnetic resonance: ^1^H-NMR, ^13^C-NMR, and 2D-heteronuclear multiple quantum coherence (HMQC).

### 3.1. Materials and Equipment

Biodiesel was made from palm oil, obtained from Research and Development (R&D) Centre of Oil and Gas Technology–LEMIGAS Indonesia. Pyrogallol, phenolphthalein indicator, methanol, ethyl acetate and sodium hydroxide were obtained from Merck. DPPH or 2,2-diphenyl-1-picrylhydrazyl, tert-butylhydroquinone, and gallic acid were obtained from Sigma-Aldrich. Methyl linoleate was obtained from Xian Plant Bio-Engineering Co., Ltd. Chloroform from Fulltime, Xian, China, ethanol 95% mallinckrodt. High Performance Liquid Chromatography (Agilent Technologies 1220 Infinity II LC) C-18 reversed phase column with ultraviolet (UV) detectors, solvent methanol:water (80:20). Thin layer chromatography with silica stationary phase from Merck. UV-VIS Spectrophotometer PG Instruments T-60, UK. pH meter Horiba LAQUAct pH meter D-71. Liquid chromatography tandem mass spectrometry (LCMS-MS) with UNIFI software, solvent: acetonitrile and using high pressure limit 18,000 psi, both MS function in the tandem MS used electron spray injection (ESI) injection type. The different between function 1 and function 2 was the collision energy of 6.00 eV and 10.00 eV respectively. The structure elucidation using JEOL 500 MHz Nuclear Magnetic Resonance, method: proton, carbon, and HMCQ with deuterated chloroform (CDCl_3_) solvent. Metrohm 873 Biodiesel Rancimat was used for the rancimat oxidation stability test.

### 3.2. Synthesis Reaction

The reaction of pyrogallol and methyl linoeate was performed in a 100 mL three-neck flask with stirrer. Stirring was kept constant at 1000 rpm at room temperature to prevent pyrogallol degradation. A solution of pyrogallol (1 mmol) in 5 mL ethyl acetate, 0.1 mg DPPH/ml in methanol and methyl linoleate (2 mmol) was prepared. Methyl linoleate was added to the flask and flowed by nitrogen gas during the reaction. A 5 mL of DPPH solution was added to a 5 mL of methyl linoleate solution in the three-neck flask and was stirred at room temperature for 30 min. The violet colour of the DPPH solution quickly turned yellow. Gradually, 5 mL pyrogallol solution was dropped into the reaction. Finally, 5 mL DPPH solution was added. The reaction was stirred for 45 min at room temperature. The product mixture was analyzed and purified using thin layer chromatography and high performace liquid chromatography (HPLC) [31]. The Pyrogallol derivative was isolated right after it was subjected to HPLC. The fractions based on retention time were collected using preparative HPLC in separate well/small tubes. The peak at retention time of 8.848 minutes fraction was further used for the LCMS-MS analysis. To confirm the molecular mass, liquid chromatography tandem mass spectra was used. The ^1^H-NMR, ^13^C-NMR, and 2D-HMQC were used for the molecular structure characterization.

### 3.3. Application to Biodiesel

For the application sections, the pyrogallol derivative product was previously prepared. It was obtained using a simple extraction method. A mixture of n-hexane, ethyl acetate, and methanol with a volume ratio of 1:1:1 was used for the separation. The non-polar n-hexane fraction was collected and evaporated using vacuum evaporation. The dry sample was in the form of oily liquid and used in further experiments as pyrogallol derivative.

#### 3.3.1. Solubility Test

The solubility limit of pyrogallol (PY), tert-butylhydroquinone (TBHQ) and gallic acid (GA) were compared with the pyrogallol derivative (PD) in biodiesel solution. An ultraviolet–visible (UV–VIS) spectrophotometer was used for the analysis. Biodiesel without antioxidants (B) was subjected to the UV-VIS spectrophotometer to obtain its wavelength versus absorbance graph to find the optimum wavelength (gallic acid 246 nm, pyrogallol 298 nm, TBHQ 268 nm, pyrogallol derivative 246 nm). The same procedure was done with different biodiesel additive mixtures: biodiesel-pyrogallol (B-PY), biodiesel-TBHQ (B-TBHQ), biodiesel-gallic acid (B-GA), and biodiesel-pyrogallol derivative (B-PD). The absorbance of biodiesel-additive mixtures at six increasing concentrations, 500 ppm, 1000 ppm, 1500 ppm, 2000 ppm, 2500 ppm, and 3000 ppm, was read at their specific maximum wavelength. The solubility limit was reached when the absorbance of the solution stop increased or remained constant with the increasing concentration. These solubility limits were used as a concentration of the additive to be added in biodiesel for the antioxidant activity test. Two-way factor analysis of variance (ANOVA) was used for the statistical analysis using SPSS software.

#### 3.3.2. Acid Number

All antioxidants were subjected to acid number analysis, each sample were mixed in the biodiesel with the concentration of 1500 ppm. The samples were labeled: biodiesel-pyrogallol (B-PY), biodiesel-TBHQ (B-TBHQ), biodiesel-gallic acid (B-GA), and biodiesel-pyrogallol derivative (B-PD) Weekly analysis were done during a four weeks storage time. A sample of 1 mL was put into an Erlenmeyer flask. Then, 50 mL of 95% neutral ethanol was added. A calibrated pH meter was prepared. Afterwards, the solution was titrated with ranging volume of a standard 0.1 N NaOH to obtain the diagram of volume of the used NaOH versus pH. After the data was plotted, the volume of NaOH required to change the pH into 7 was calculated using the curves’ equation. The acid values of the samples were calculated using method from Indonesian National Standard for oil and lipid SNI 01-3555-1998 [32].

#### 3.3.3. Viscosity

Viscosity is the ratio of shearing stress to the gradient of the liquid’s velocity. The viscosity value is commonly used as a marker of biodiesel’s oxidation due to the formation of secondary oxidation products of oil polymerization [21]. In this experiment all antioxidant samples were measured at 40 °C using Otswald tube equipment [33].

#### 3.3.4. Oxidation Stability Test (Rancimat Test)

The biodiesel oxidation stability test was performed by using a Metrohm 873 Biodiesel Rancimat instrument in accordance to the standard test method EN 14112 [21,34,35]. This method accelerates the aging process of the biodiesel sample in the presence of heat of 110 °C and a continues air flow with the rate of 10 L/h. In this condition, biodiesel was oxidizes producing volatile, water-soluble oxidation products such as short chain carboxylic acid like formic acid. This event is detected by the increasing of biodiesel’s conductivity. The equipment measures the time required (h) until the oxidation occurs at the high rate or also known as induction time/induction period (IP). 

## 4. Conclusions

The reaction produced a non-polar pyrogallol derivative molecule methyl (10*E*,12*E*)-9-(2,6-dihydroxyphenoxy)octadeca-10,12-dienoate and its isomer methyl (9*E*,11*E*)-13-(2,6-dihydroxyphenoxy)octadeca-9,11-dienoate with 12.86% yield and selectivity of 21.05% on the basis of pyrogallol. The product mixture has a higher solubility in biodiesel compared to pyrogallol, TBHQ, and gallic acid. The antioxidant analysis of the pyrogallol derivative on the acid number, viscosity, and Rancimat oxidation stability test resulted in potential use for antioxidant additive for biodiesel.

## Figures and Tables

**Table 1:** 2D-heteronuclear multiple quantum coherence (HMQC) correlation of ^13^C nuclear magnetic resonance (NMR) and ^1^H-NMR signals for pyrogallol derivative.

Carbon	Chemical Shift (ppm)		^1^H-NMR Signal
	^13^C-NMR	^1^H-NMR	
**a**	14.271	0.873	t, 3H
b	22.766	1.225–1.307	m, 2H
c	25.809		m, 2H
d	29.303		m, 2H
l	31.712		m, 2H
m	29.773		m, 2H
n	29.533		m, 2H
o	27.373		m, 2H
p	25.127		m, 2H
e	27.373	2.025	q, 2H
k	27.373		q, 2H
q	34.303	2.290	t, 2H
f	128.092	5.288–5.383	m, 1H
g	128.227		m, 1H
h	130.242		m, 1H
i	130.415		m, 1H
r	174.656	No proton	-
s	51.696	3.653	s, 3H
u	108.175	6.435	d, 1H
w			d, 1H
y	144.362	No proton	-
v	120.308	6.634	t, 1H
j	58.742	3.682–3.724	q, 1H
x	Not detected	Not detected	s, OH
t			s, OH

## Supplementary Materials

The supplementary materials are available online. Figure S1. ^1^H-NMR, ^13^C-NMR, and 2D-HMQC spectrum. Figure S2. 2D-HMQC correlation between ^1^H-NMR and ^13^C-NMR of carbon (j). Figure S3. 2D-HMQC correlation between ^1^H-NMR and ^13^C-NMR of proton and carbon (t,x,w,u), and correlation between ^1^H-NMR and ^13^C-NMR of proton and carbon (f,g,h,i).
