# Supplementary material for: Solubility and Antioxidant Potential of a Pyrogallol Derivative for Biodiesel Additive
Source: Molecules. 2019 Jul 2;24(13):2439. doi: 10.3390/molecules24132439 (PMC6651424; doi:10.3390/molecules24132439)
Supplement: Supplementary file 1 [file molecules-24-02439-s001.zip › molecules-522407 supplementary .pdf]

Supplementary Materials

Figure S1. ^{1}H-NMR, ^{13}C-NMR, and 2D-HMQC spectrum.

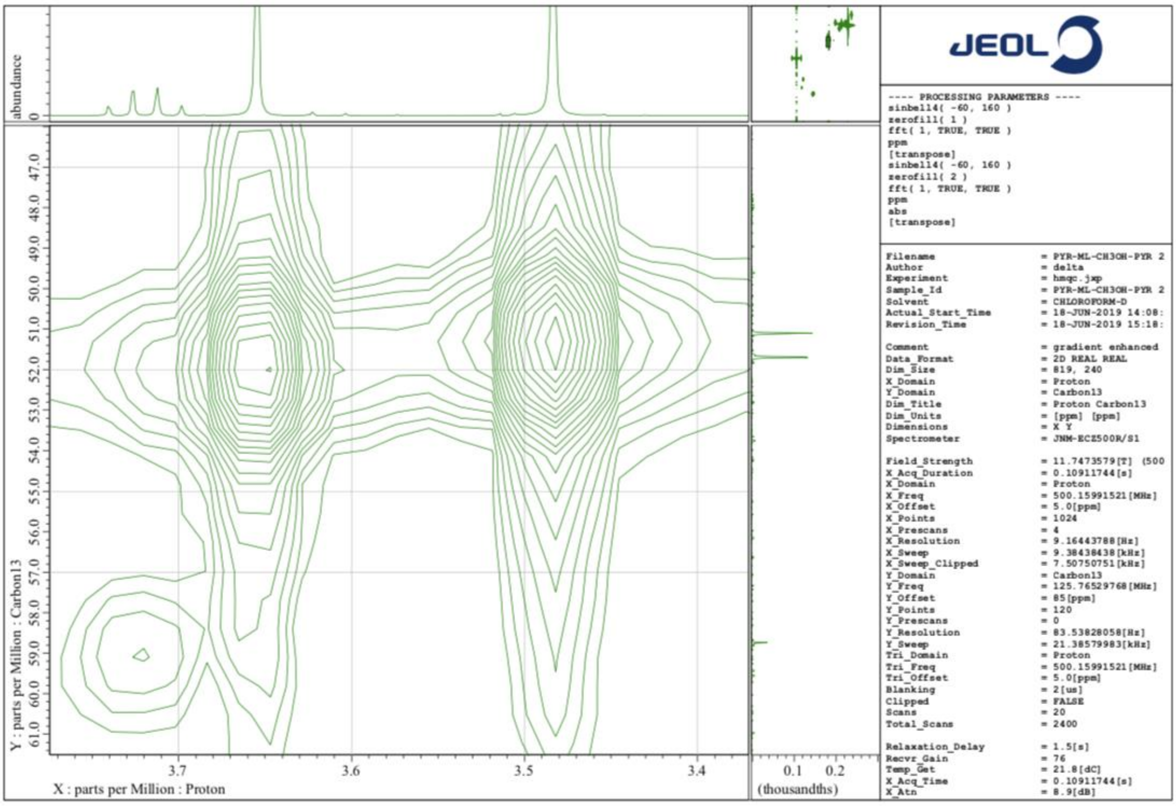

Figure 2. 2D-HMQC correlation between ^{1}H-NMR and ^{13}C-NMR of carbon (j).

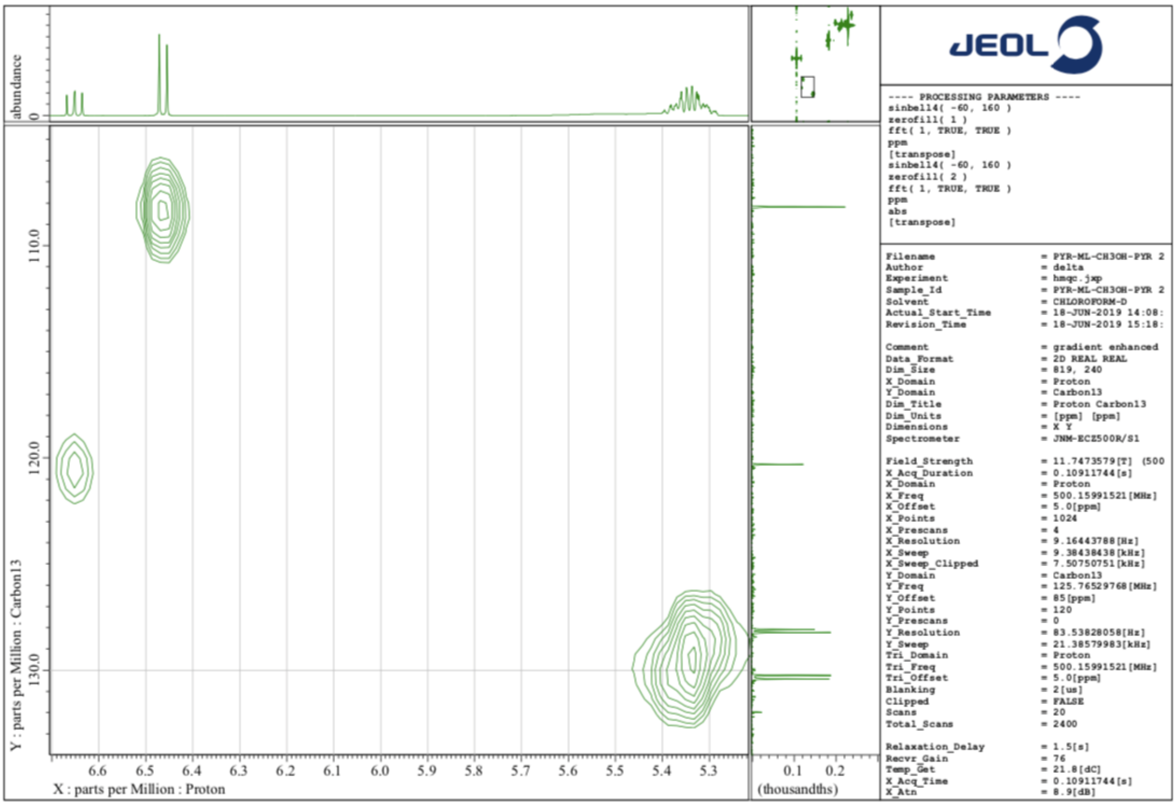

Figure 3. 2D-HMQC correlation between ^{1}H-NMR and ^{13}C-NMR of proton and carbon (t,x,w,u), and correlation between ^{1}H-NMR and ^{13}C-NMR of proton and carbon (f,g,h,i).
