# Supplementary material for: Solubility and Antioxidant Potential of a Pyrogallol Derivative for Biodiesel Additive
Source: Molecules. 2019 Jul 2;24(13):2439. doi: 10.3390/molecules24132439 (PMC6651424; doi:10.3390/molecules24132439)
Supplement: Supplementary file 1 [file molecules-24-02439-s001.zip › Figure S1 1H-NMR, 13C-NMR, and 2D-HMQC spectrum.pdf]

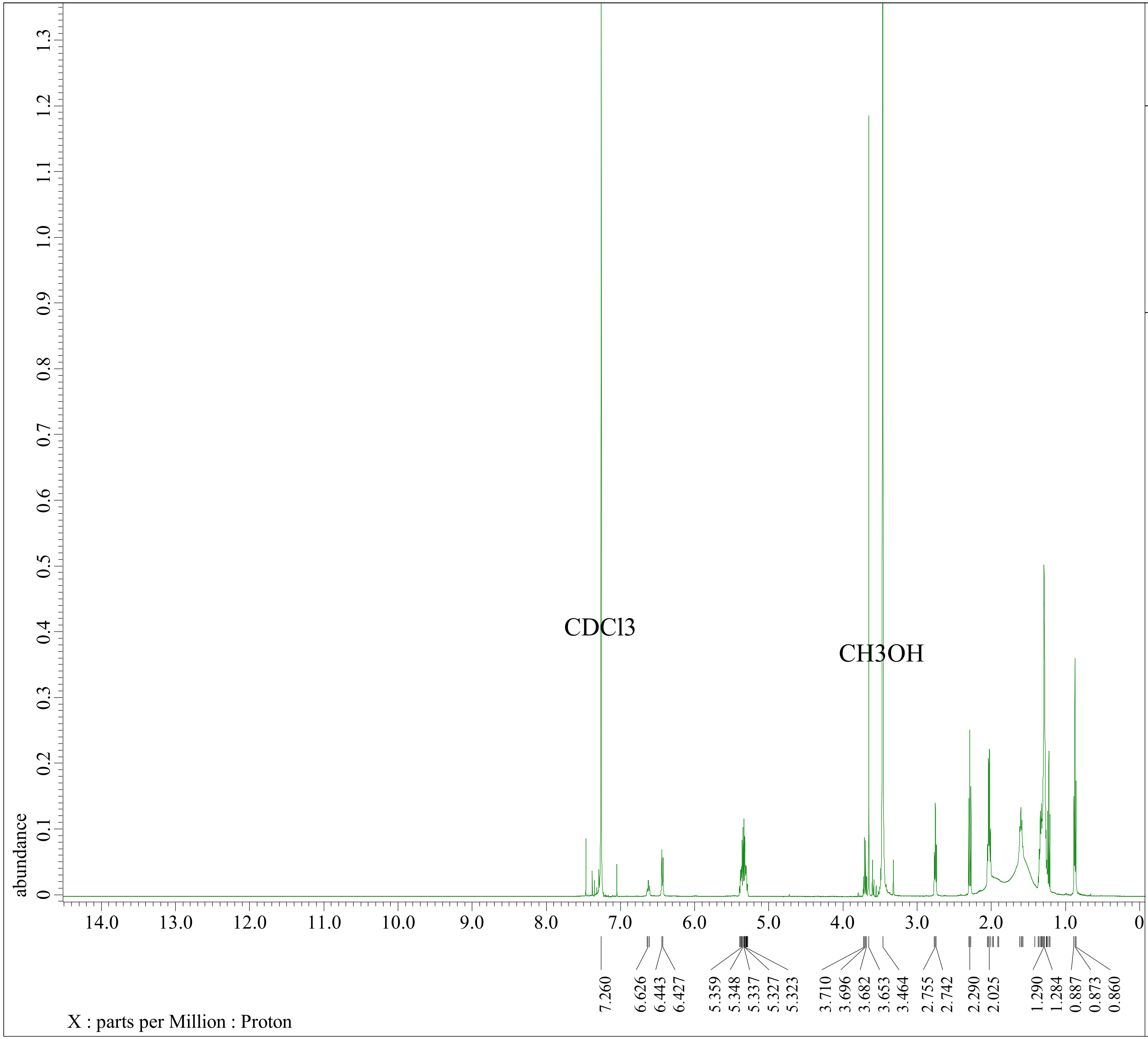

JEOL

---- PROCESSING PARAMETERS ----
sexp(0.2[Hz], 0.0[s])
trapezoid(0[%], 0[%], 80[%], 100[%])
zerofill(1)
fft(1, TRUE, TRUE)
machinephase
ppm
phase(3.34008, 0, 48.29086[%])

Derived from: PYR-METLIN-CH3OH-PYR_Proton_cop

Filename	= PYR-METLIN-CH3OH-PYR_
Author	= delta
Experiment	= proton.jxp
Sample_Id	= PYR-METLIN-CH3OH-PYR
Solvent	= CHLOROFORM-D
Actual_Start_Time	= 11-JUN-2019 11:36:48
Revision_Time	= 12-JUN-2019 16:04:50
Comment	= single_pulse
Data_Format	= 1D COMPLEX
Dim_Size	= 13107
X_Domain	= Proton
Dim_Title	= Proton
Dim_Units	= [ppm]
Dimensions	= X
Spectrometer	= JNM-ECZ500R/S1
Field_Strength	= 11.7473579[T] (500[MH
X_Acq_Duration	= 0[s]
X_Domain	= Proton
X_Freq	= 500.15991521[MHz]
X_Offset	= 7.0[ppm]
X_Points	= 16384
X_Prescans	= 1
X_Resolution	= 0.57277737[Hz]
X_Sweep	= 9.38438438[kHz]
X_Sweep_Clipped	= 7.50750751[kHz]
Irr_Domain	= Proton
Irr_Freq	= 500.15991521[MHz]
Irr_Offset	= 5.0[ppm]
Tri_Domain	= Proton
Tri_Freq	= 500.15991521[MHz]
Tri_Offset	= 5.0[ppm]
Blanking	= 2[us]
Clipped	= FALSE
Incomplete_Copy	= TRUE
Scans	= 32
Total_Scans	= 32
Relaxation_Delay	= 5[s]
Recvr_Gain	= 46
Temp_Get	= 21.4[dC]
X_90_Width	= 8[us]
X_Acq_Time	= 1.74587904[s]
X_Angle	= 45[deg]
X_Atn	= 8.9[dB]
X_Pulse	= 4[us]
Irr_Mode	= Off

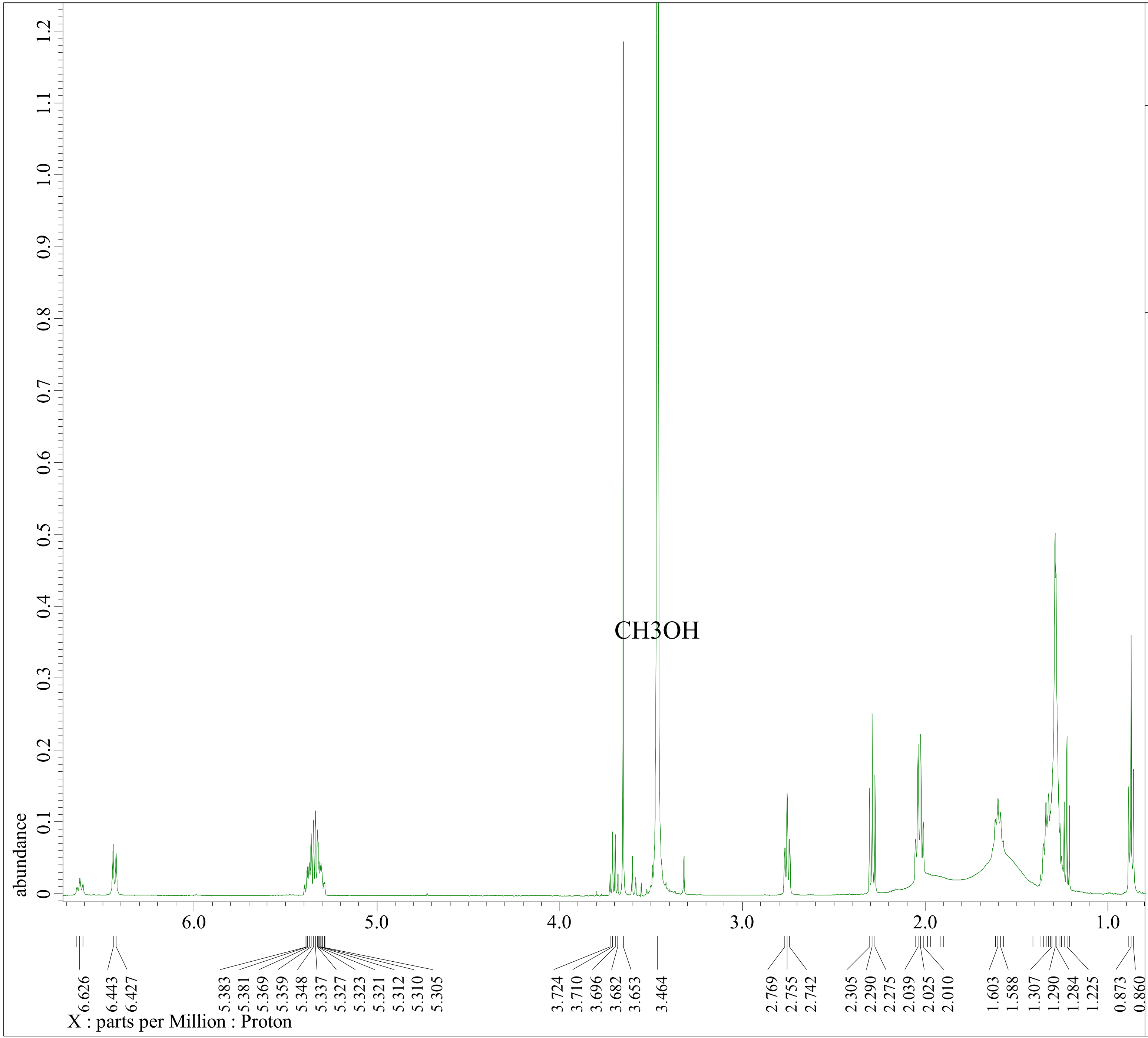


```
---- PROCESSING PARAMETERS ----
sexp( 0.2[Hz], 0.0[s] )
trapezoid( 0[%], 0[%], 80[%], 100[%] )
zerofill( 1 )
fft( 1, TRUE, TRUE )
machinephase
ppm
phase( 3.34008, 0, 48.29086[%] )

Derived from: PYR-METLIN-CH3OH-PYR_Proton_cop
```

```
Filename              = PYR-METLIN-CH3OH-PYR_
Author                = delta
Experiment            = proton.jxp
Sample_Id             = PYR-METLIN-CH3OH-PYR
Solvent               = CHLOROFORM-D
Actual_Start_Time     = 11-JUN-2019 11:36:48
Revision_Time         = 12-JUN-2019 16:04:50

Comment               = single_pulse
Data_Format           = 1D COMPLEX
Dim_Size              = 13107
X_Domain              = Proton
Dim_Title             = Proton
Dim_Units             = [ppm]
Dimensions            = X
Spectrometer          = JNM-ECZ500R/S1

Field_Strength        = 11.7473579[T] (500[MH
X_Acq_Duration        = 0[s]
X_Domain              = Proton
X_Freq                = 500.15991521[MHz]
X_Offset              = 7.0[ppm]
X_Points              = 16384
X_Prescans            = 1
X_Resolution          = 0.57277737[Hz]
X_Sweep               = 9.38438438[kHz]
X_Sweep_Clipped       = 7.50750751[kHz]
Irr_Domain            = Proton
Irr_Freq              = 500.15991521[MHz]
Irr_Offset            = 5.0[ppm]
Tri_Domain            = Proton
Tri_Freq              = 500.15991521[MHz]
Tri_Offset            = 5.0[ppm]
Blanking              = 2[us]
Clipped               = FALSE
Incomplete_Copy       = TRUE
Scans                 = 32
Total_Scans           = 32

Relaxation_Delay      = 5[s]
Recvr_Gain            = 46
Temp_Get              = 21.4[dC]
X_90_Width            = 8[us]
X_Acq_Time            = 1.74587904[s]
X_Angle               = 45[deg]
X_Atn                 = 8.9[dB]
X_Pulse               = 4[us]
Irr_Mode              = Off
```

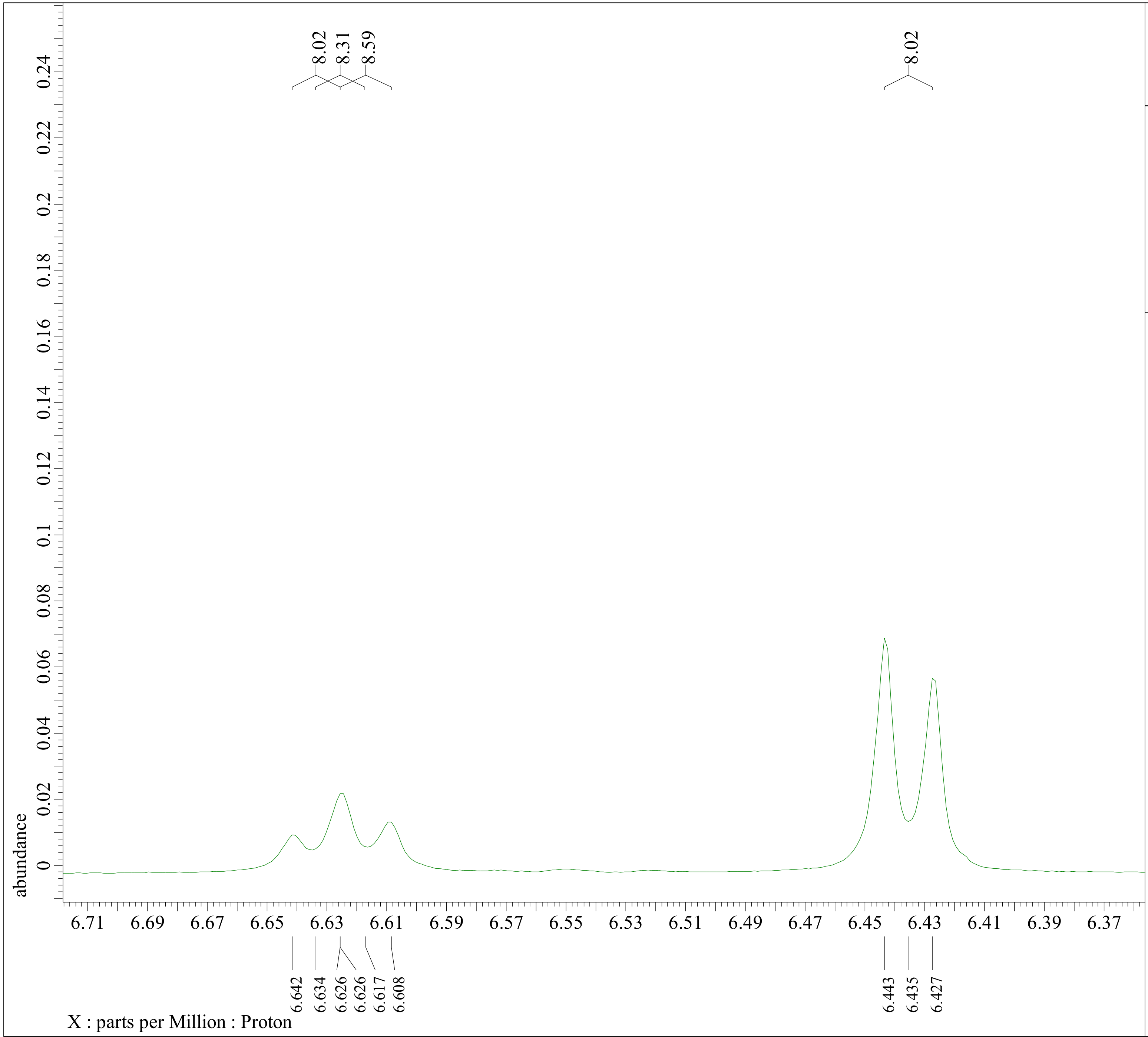


```
---- PROCESSING PARAMETERS ----
sexp( 0.2[Hz], 0.0[s] )
trapezoid( 0[%], 0[%], 80[%], 100[%] )
zerofill( 1 )
fft( 1, TRUE, TRUE )
machinephase
ppm
phase( 3.34008, 0, 48.29086[%] )

Derived from: PYR-METLIN-CH3OH-PYR_Proton_cop
```

```
Filename              = PYR-METLIN-CH3OH-PYR_
Author                = delta
Experiment            = proton.jxp
Sample_Id             = PYR-METLIN-CH3OH-PYR
Solvent               = CHLOROFORM-D
Actual_Start_Time     = 11-JUN-2019 11:36:48
Revision_Time         = 12-JUN-2019 16:08:18

Comment               = single_pulse
Data_Format           = 1D COMPLEX
Dim_Size              = 13107
X_Domain              = Proton
Dim_Title             = Proton
Dim_Units             = [ppm]
Dimensions            = X
Spectrometer          = JNM-ECZ500R/S1

Field_Strength        = 11.7473579[T] (500[MH
X_Acq_Duration        = 0[s]
X_Domain              = Proton
X_Freq                = 500.15991521[MHz]
X_Offset              = 7.0[ppm]
X_Points              = 16384
X_Prescans            = 1
X_Resolution          = 0.57277737[Hz]
X_Sweep               = 9.38438438[kHz]
X_Sweep_Clipped       = 7.50750751[kHz]
Irr_Domain            = Proton
Irr_Freq              = 500.15991521[MHz]
Irr_Offset            = 5.0[ppm]
Tri_Domain            = Proton
Tri_Freq              = 500.15991521[MHz]
Tri_Offset            = 5.0[ppm]
Blanking              = 2[us]
Clipped               = FALSE
Incomplete_Copy       = TRUE
Scans                 = 32
Total_Scans           = 32

Relaxation_Delay      = 5[s]
Recvr_Gain            = 46
Temp_Get              = 21.4[dC]
X_90_Width            = 8[us]
X_Acq_Time            = 1.74587904[s]
X_Angle               = 45[deg]
X_Atn                 = 8.9[dB]
X_Pulse               = 4[us]
Irr_Mode              = Off
```

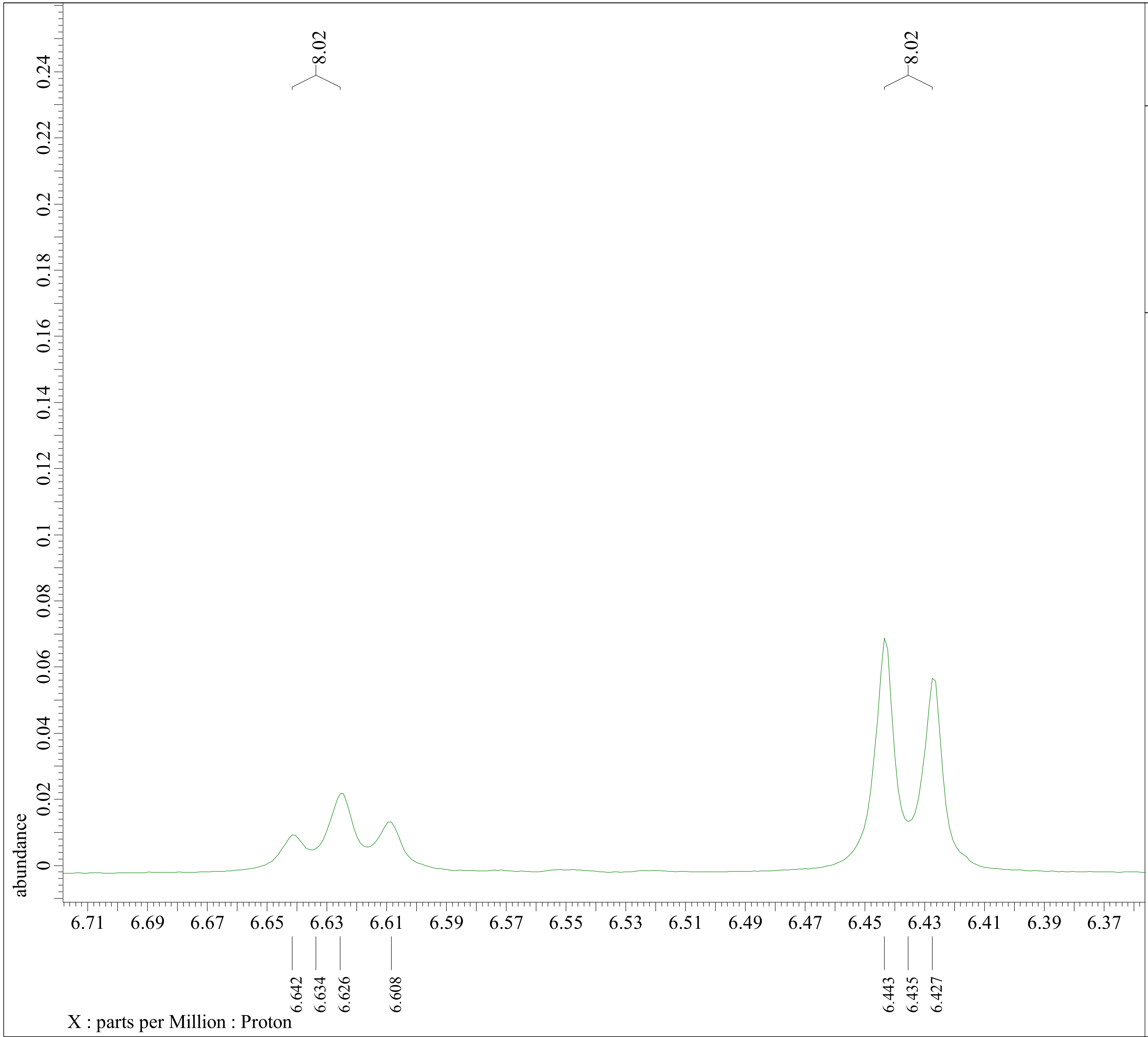

JEOL

```
---- PROCESSING PARAMETERS ----
sexp( 0.2[Hz], 0.0[s] )
trapezoid( 0[%], 0[%], 80[%], 100[%] )
zerofill( 1 )
fft( 1, TRUE, TRUE )
machinephase
ppm
phase( 3.34008, 0, 48.29086[%] )

Derived from: PYR-METLIN-CH3OH-PYR_Proton_cop
```

```
Filename             = PYR-METLIN-CH3OH-PYR_
Author               = delta
Experiment           = proton.jxp
Sample_Id            = PYR-METLIN-CH3OH-PYR
Solvent              = CHLOROFORM-D
Actual_Start_Time    = 11-JUN-2019 11:36:48
Revision_Time        = 12-JUN-2019 16:08:59

Comment              = single_pulse
Data_Format          = 1D COMPLEX
Dim_Size             = 13107
X_Domain             = Proton
Dim_Title            = Proton
Dim_Units            = [ppm]
Dimensions           = X
Spectrometer         = JNM-ECZ500R/S1

Field_Strength       = 11.7473579[T] (500[MH
X_Acq_Duration       = 0[s]
X_Domain             = Proton
X_Freq               = 500.15991521[MHz]
X_Offset             = 7.0[ppm]
X_Points             = 16384
X_Prescans           = 1
X_Resolution         = 0.57277737[Hz]
X_Sweep              = 9.38438438[kHz]
X_Sweep_Clipped      = 7.50750751[kHz]
Irr_Domain           = Proton
Irr_Freq             = 500.15991521[MHz]
Irr_Offset           = 5.0[ppm]
Tri_Domain           = Proton
Tri_Freq             = 500.15991521[MHz]
Tri_Offset           = 5.0[ppm]
Blanking             = 2[us]
Clipped              = FALSE
Incomplete_Copy      = TRUE
Scans                = 32
Total_Scans          = 32

Relaxation_Delay     = 5[s]
Recvr_Gain           = 46
Temp_Get             = 21.4[dC]
X_90_Width           = 8[us]
X_Acq_Time           = 1.74587904[s]
X_Angle              = 45[deg]
X_Atn                = 8.9[dB]
X_Pulse              = 4[us]
Irr_Mode             = Off
```

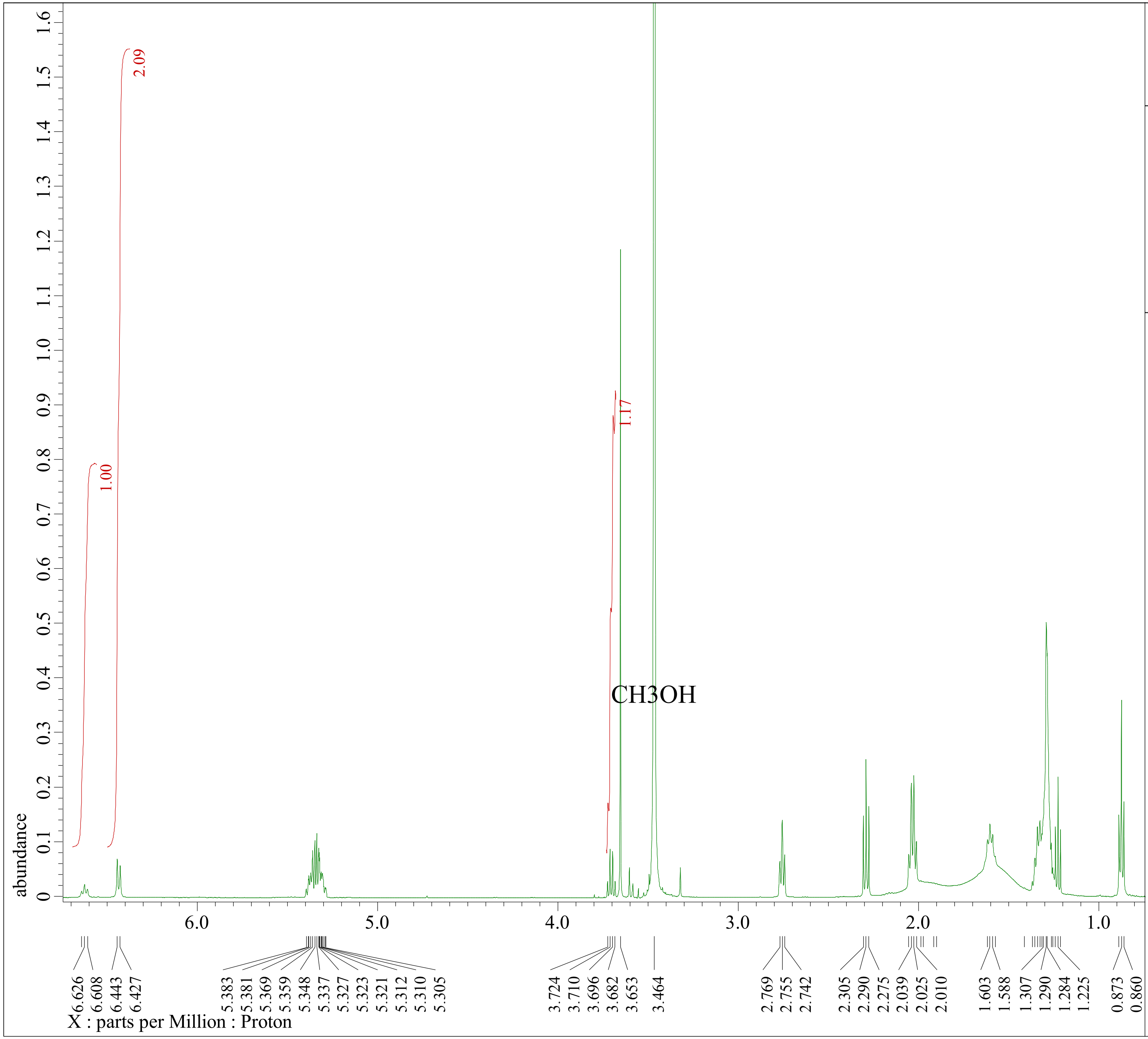

---- PROCESSING PARAMETERS ----
sexp(0.2[Hz], 0.0[s])
trapezoid(0[%], 0[%], 80[%], 100[%])
zerofill(1)
fft(1, TRUE, TRUE)
machinephase
ppm
phase(3.34008, 0, 48.29086[%])

Derived from: PYR-METLIN-CH3OH-PYR_Proton_cop

Filename	= PYR-METLIN-CH3OH-PYR_
Author	= delta
Experiment	= proton.jxp
Sample_Id	= PYR-METLIN-CH3OH-PYR
Solvent	= CHLOROFORM-D
Actual_Start_Time	= 11-JUN-2019 11:36:48
Revision_Time	= 12-JUN-2019 16:20:50
Comment	= single_pulse
Data_Format	= 1D COMPLEX
Dim_Size	= 13107
X_Domain	= Proton
Dim_Title	= Proton
Dim_Units	= [ppm]
Dimensions	= X
Spectrometer	= JNM-ECZ500R/S1
Field_Strength	= 11.7473579[T] (500[MH
X_Acq_Duration	= 0[s]
X_Domain	= Proton
X_Freq	= 500.15991521[MHz]
X_Offset	= 7.0[ppm]
X_Points	= 16384
X_Prescans	= 1
X_Resolution	= 0.57277737[Hz]
X_Sweep	= 9.38438438[kHz]
X_Sweep_Clipped	= 7.50750751[kHz]
Irr_Domain	= Proton
Irr_Freq	= 500.15991521[MHz]
Irr_Offset	= 5.0[ppm]
Tri_Domain	= Proton
Tri_Freq	= 500.15991521[MHz]
Tri_Offset	= 5.0[ppm]
Blanking	= 2[us]
Clipped	= FALSE
Incomplete_Copy	= TRUE
Scans	= 32
Total_Scans	= 32
Relaxation_Delay	= 5[s]
Recvr_Gain	= 46
Temp_Get	= 21.4[dC]
X_90_Width	= 8[us]
X_Acq_Time	= 1.74587904[s]
X_Angle	= 45[deg]
X_Atn	= 8.9[dB]
X_Pulse	= 4[us]
Irr_Mode	= Off

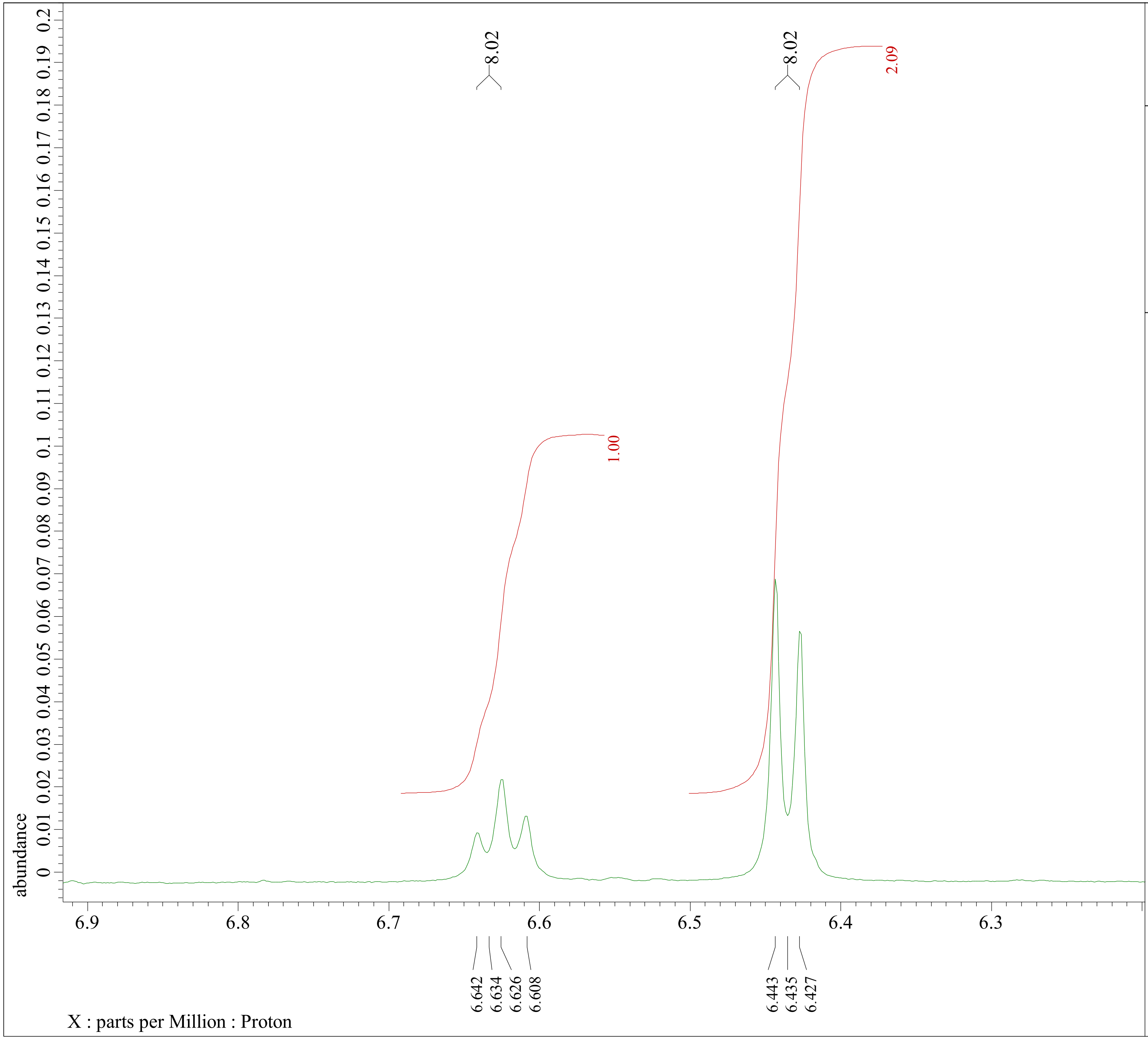


```
---- PROCESSING PARAMETERS ----
sexp( 0.2[Hz], 0.0[s] )
trapezoid( 0[%], 0[%], 80[%], 100[%] )
zerofill( 1 )
fft( 1, TRUE, TRUE )
machinephase
ppm
phase( 3.34008, 0, 48.29086[%] )

Derived from: PYR-METLIN-CH3OH-PYR_Proton_cop
```

```
Filename              = PYR-METLIN-CH3OH-PYR_
Author                = delta
Experiment            = proton.jxp
Sample_Id             = PYR-METLIN-CH3OH-PYR
Solvent               = CHLOROFORM-D
Actual_Start_Time     = 11-JUN-2019 11:36:48
Revision_Time         = 12-JUN-2019 16:22:08

Comment               = single_pulse
Data_Format           = 1D COMPLEX
Dim_Size              = 13107
X_Domain              = Proton
Dim_Title             = Proton
Dim_Units             = [ppm]
Dimensions            = X
Spectrometer          = JNM-ECZ500R/S1

Field_Strength        = 11.7473579[T] (500[MH
X_Acq_Duration        = 0[s]
X_Domain              = Proton
X_Freq                = 500.15991521[MHz]
X_Offset              = 7.0[ppm]
X_Points              = 16384
X_Prescans            = 1
X_Resolution          = 0.57277737[Hz]
X_Sweep               = 9.38438438[kHz]
X_Sweep_Clipped       = 7.50750751[kHz]
Irr_Domain            = Proton
Irr_Freq              = 500.15991521[MHz]
Irr_Offset            = 5.0[ppm]
Tri_Domain            = Proton
Tri_Freq              = 500.15991521[MHz]
Tri_Offset            = 5.0[ppm]
Blanking              = 2[us]
Clipped               = FALSE
Incomplete_Copy       = TRUE
Scans                 = 32
Total_Scans           = 32

Relaxation_Delay      = 5[s]
Recvr_Gain            = 46
Temp_Get              = 21.4[dC]
X_90_Width            = 8[us]
X_Acq_Time            = 1.74587904[s]
X_Angle               = 45[deg]
X_Atn                 = 8.9[dB]
X_Pulse               = 4[us]
Irr_Mode              = Off
```

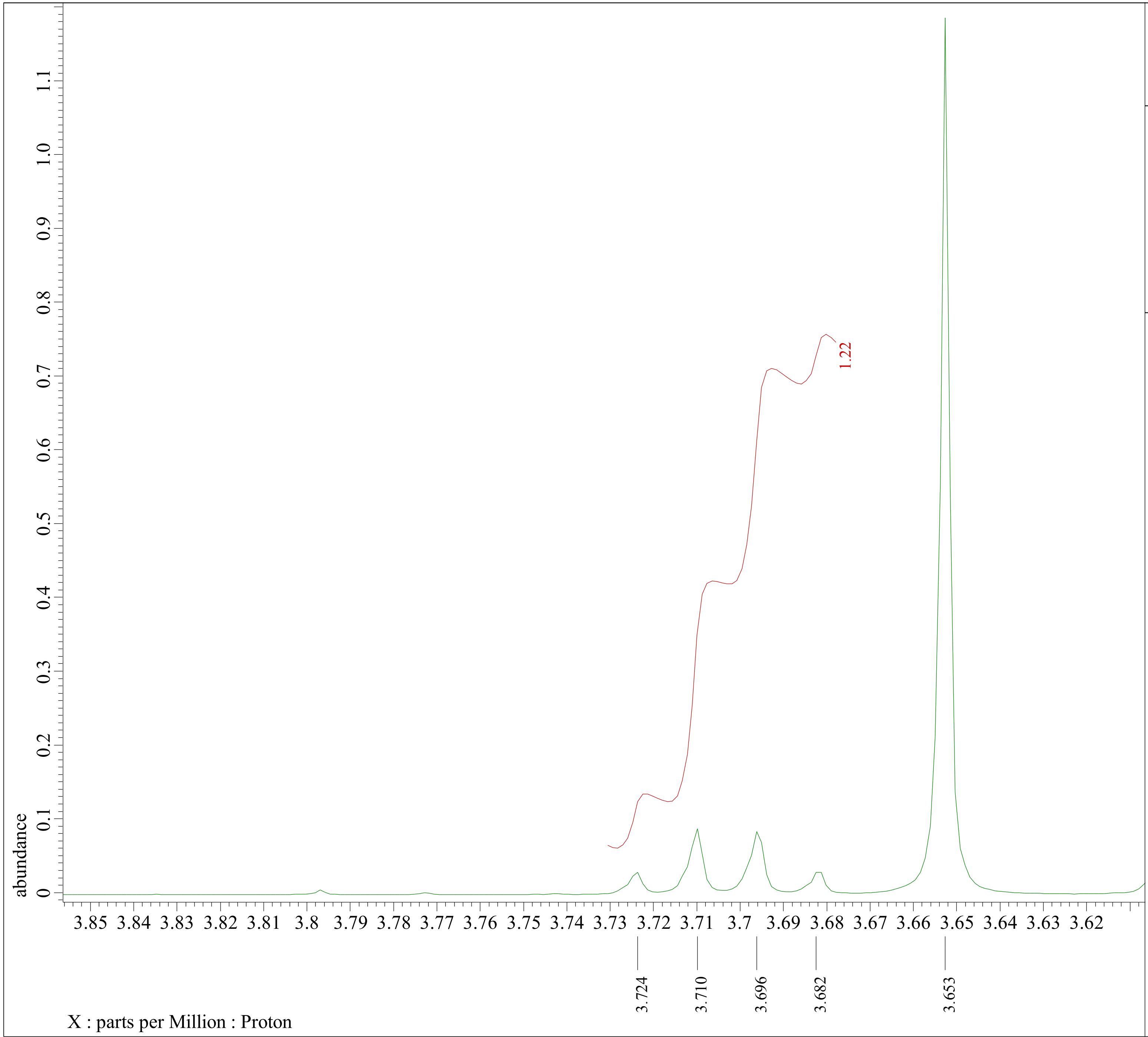

JEOL

```
---- PROCESSING PARAMETERS ----
sexp( 0.2[Hz], 0.0[s] )
trapezoid( 0[%], 0[%], 80[%], 100[%] )
zerofill( 1 )
fft( 1, TRUE, TRUE )
machinephase
ppm
phase( 3.34008, 0, 48.29086[%] )

Derived from: PYR-METLIN-CH3OH-PYR_Proton_cop

Filename             = PYR-METLIN-CH3OH-PYR_
Author               = delta
Experiment           = proton.jxp
Sample_Id            = PYR-METLIN-CH3OH-PYR
Solvent              = CHLOROFORM-D
Actual_Start_Time    = 11-JUN-2019 11:36:48
Revision_Time        = 12-JUN-2019 16:23:29

Comment              = single_pulse
Data_Format          = 1D COMPLEX
Dim_Size             = 13107
X_Domain             = Proton
Dim_Title            = Proton
Dim_Units            = [ppm]
Dimensions           = X
Spectrometer         = JNM-ECZ500R/S1

Field_Strength       = 11.7473579[T] (500[MH
X_Acq_Duration       = 0[s]
X_Domain             = Proton
X_Freq               = 500.15991521[MHz]
X_Offset             = 7.0[ppm]
X_Points             = 16384
X_Prescans           = 1
X_Resolution         = 0.57277737[Hz]
X_Sweep              = 9.38438438[kHz]
X_Sweep_Clipped      = 7.50750751[kHz]
Irr_Domain           = Proton
Irr_Freq             = 500.15991521[MHz]
Irr_Offset           = 5.0[ppm]
Tri_Domain           = Proton
Tri_Freq             = 500.15991521[MHz]
Tri_Offset           = 5.0[ppm]
Blanking             = 2[us]
Clipped              = FALSE
Incomplete_Copy      = TRUE
Scans                = 32
Total_Scans          = 32

Relaxation_Delay     = 5[s]
Recvr_Gain           = 46
Temp_Get             = 21.4[dC]
X_90_Width           = 8[us]
X_Acq_Time           = 1.74587904[s]
X_Angle              = 45[deg]
X_Atn                = 8.9[dB]
X_Pulse              = 4[us]
Irr_Mode             = Off
```

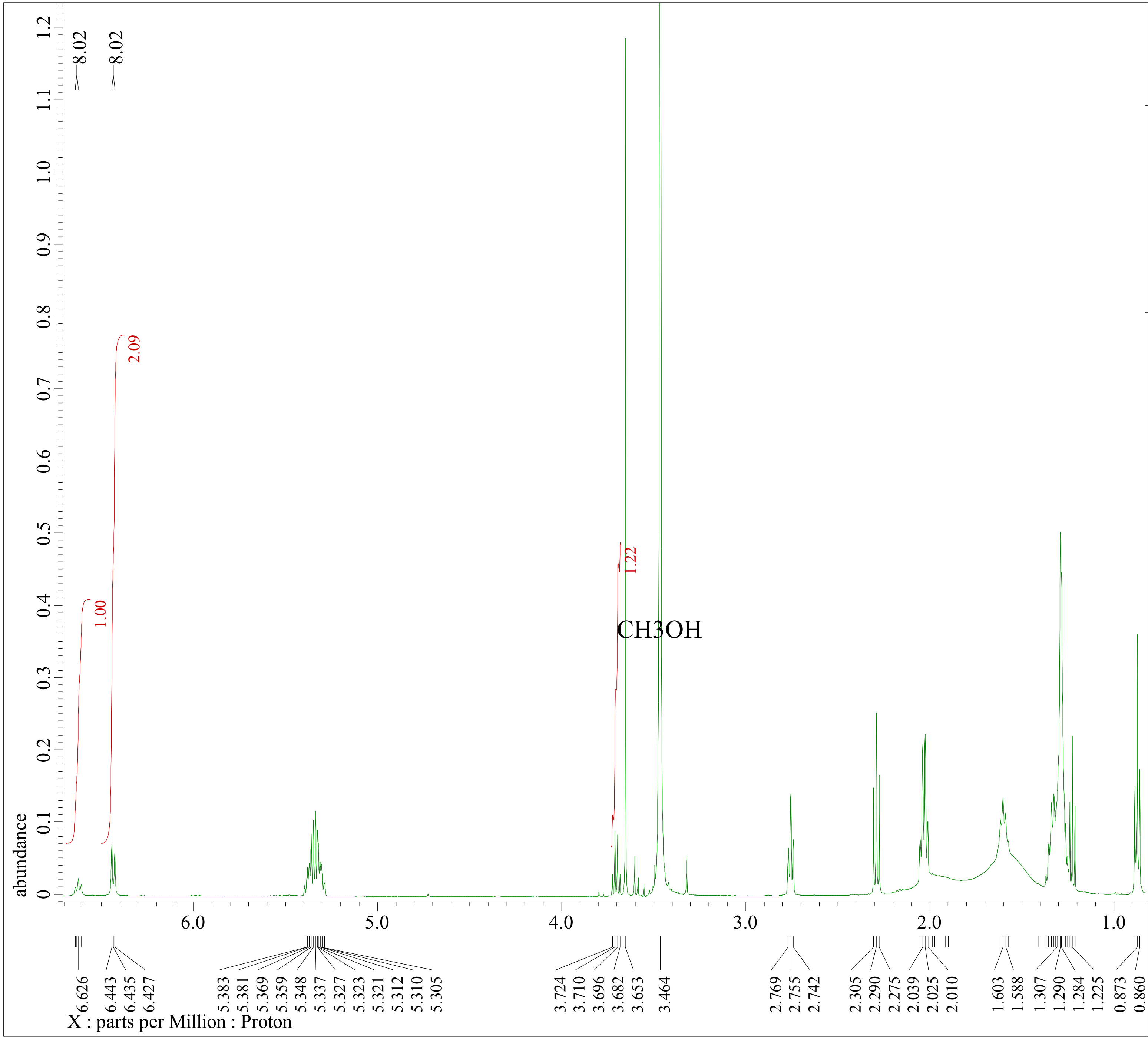


```
---- PROCESSING PARAMETERS ----
sexp( 0.2[Hz], 0.0[s] )
trapezoid( 0[%], 0[%], 80[%], 100[%] )
zerofill( 1 )
fft( 1, TRUE, TRUE )
machinephase
ppm
phase( 3.34008, 0, 48.29086[%] )

Derived from: PYR-METLIN-CH3OH-PYR_Proton_cop
```

```
Filename              = PYR-METLIN-CH3OH-PYR_
Author                = delta
Experiment            = proton.jxp
Sample_Id             = PYR-METLIN-CH3OH-PYR
Solvent               = CHLOROFORM-D
Actual_Start_Time     = 11-JUN-2019 11:36:48
Revision_Time         = 12-JUN-2019 16:23:29

Comment               = single_pulse
Data_Format           = 1D COMPLEX
Dim_Size              = 13107
X_Domain              = Proton
Dim_Title             = Proton
Dim_Units             = [ppm]
Dimensions            = X
Spectrometer          = JNM-ECZ500R/S1

Field_Strength        = 11.7473579[T] (500[MH
X_Acq_Duration        = 0[s]
X_Domain              = Proton
X_Freq                = 500.15991521[MHz]
X_Offset              = 7.0[ppm]
X_Points              = 16384
X_Prescans            = 1
X_Resolution          = 0.57277737[Hz]
X_Sweep               = 9.38438438[kHz]
X_Sweep_Clipped       = 7.50750751[kHz]
Irr_Domain            = Proton
Irr_Freq              = 500.15991521[MHz]
Irr_Offset            = 5.0[ppm]
Tri_Domain            = Proton
Tri_Freq              = 500.15991521[MHz]
Tri_Offset            = 5.0[ppm]
Blanking              = 2[us]
Clipped               = FALSE
Incomplete_Copy       = TRUE
Scans                 = 32
Total_Scans           = 32

Relaxation_Delay      = 5[s]
Recvr_Gain            = 46
Temp_Get              = 21.4[dC]
X_90_Width            = 8[us]
X_Acq_Time            = 1.74587904[s]
X_Angle               = 45[deg]
X_Atn                 = 8.9[dB]
X_Pulse               = 4[us]
Irr_Mode              = Off
```

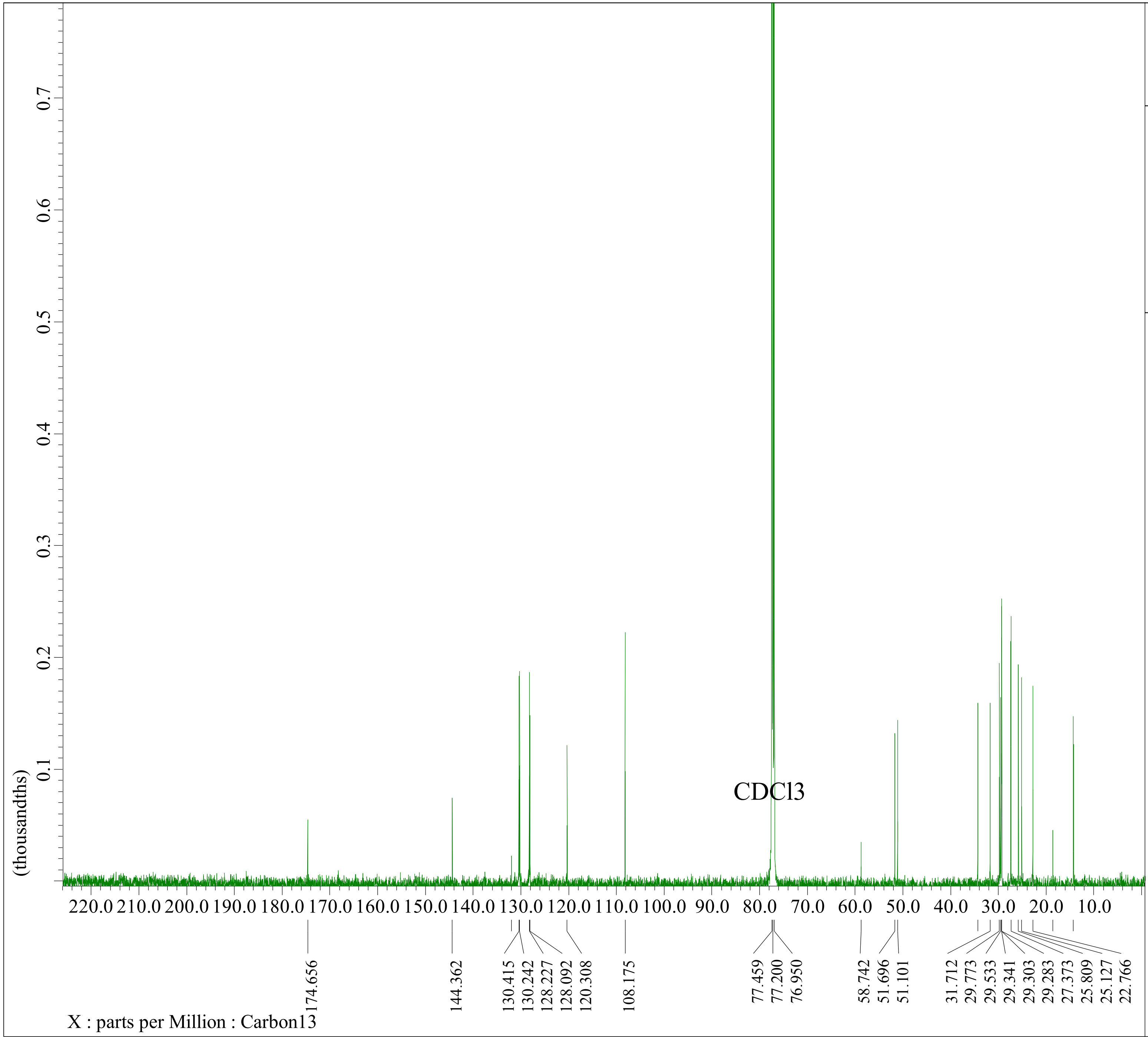


```
---- PROCESSING PARAMETERS ----
sexp( 2.0[Hz], 0.0[s] )
trapezoid( 0[%], 0[%], 80[%], 100[%] )
zerofill( 1 )
fft( 1, TRUE, TRUE )
machinephase
ppm

Filename                    = PYR-ML-CH3OH-PYR 2
Author                      = delta
Experiment                  = carbon.jxp
Sample_Id                   = PYR-ML-CH3OH-PYR 2
Solvent                     = CHLOROFORM-D
Actual_Start_Time           = 18-JUN-2019 09:34:
Revision_Time               = 18-JUN-2019 14:58:

Comment                     = single pulse decou
Data_Format                 = 1D COMPLEX
Dim_Size                    = 26214
X_Domain                    = Carbon13
Dim_Title                   = Carbon13
Dim_Units                   = [ppm]
Dimensions                  = X
Spectrometer                = JNM-ECZ500R/S1

Field_Strength              = 11.7473579[T] (500
X_Acq_Duration              = 0.82837504[s]
X_Domain                    = Carbon13
X_Freq                      = 125.76529768[MHz]
X_Offset                    = 100[ppm]
X_Points                    = 32768
X_Prescans                  = 4
X_Resolution                = 1.20718268[Hz]
X_Sweep                     = 39.55696203[kHz]
X_Sweep_Clipped             = 31.64556962[kHz]
Irr_Domain                  = Proton
Irr_Freq                    = 500.15991521[MHz]
Irr_Offset                  = 5.0[ppm]
Blanking                    = 2[us]
Clipped                     = FALSE
Scans                       = 5718
Total_Scans                 = 5718

Relaxation_Delay            = 2[s]
Recvr_Gain                  = 36
Temp_Get                    = 21.5[dC]
X_90_Width                  = 11.3[us]
X_Acq_Time                  = 0.82837504[s]
X_Angle                     = 30[deg]
X_Atn                       = 11[dB]
X_Pulse                     = 3.76666667[us]
Irr_Atn_Dec                 = 30.11[dB]
Irr_Atn_Dec_Calc            = 30.11[dB]
Irr_Atn_Dec_Default_Calc    = 30.11[dB]
Irr_Atn_Noe                 = 30.11[dB]
Irr_Dec_Bandwidth_Hz        = 5.97826087[kHz]
```

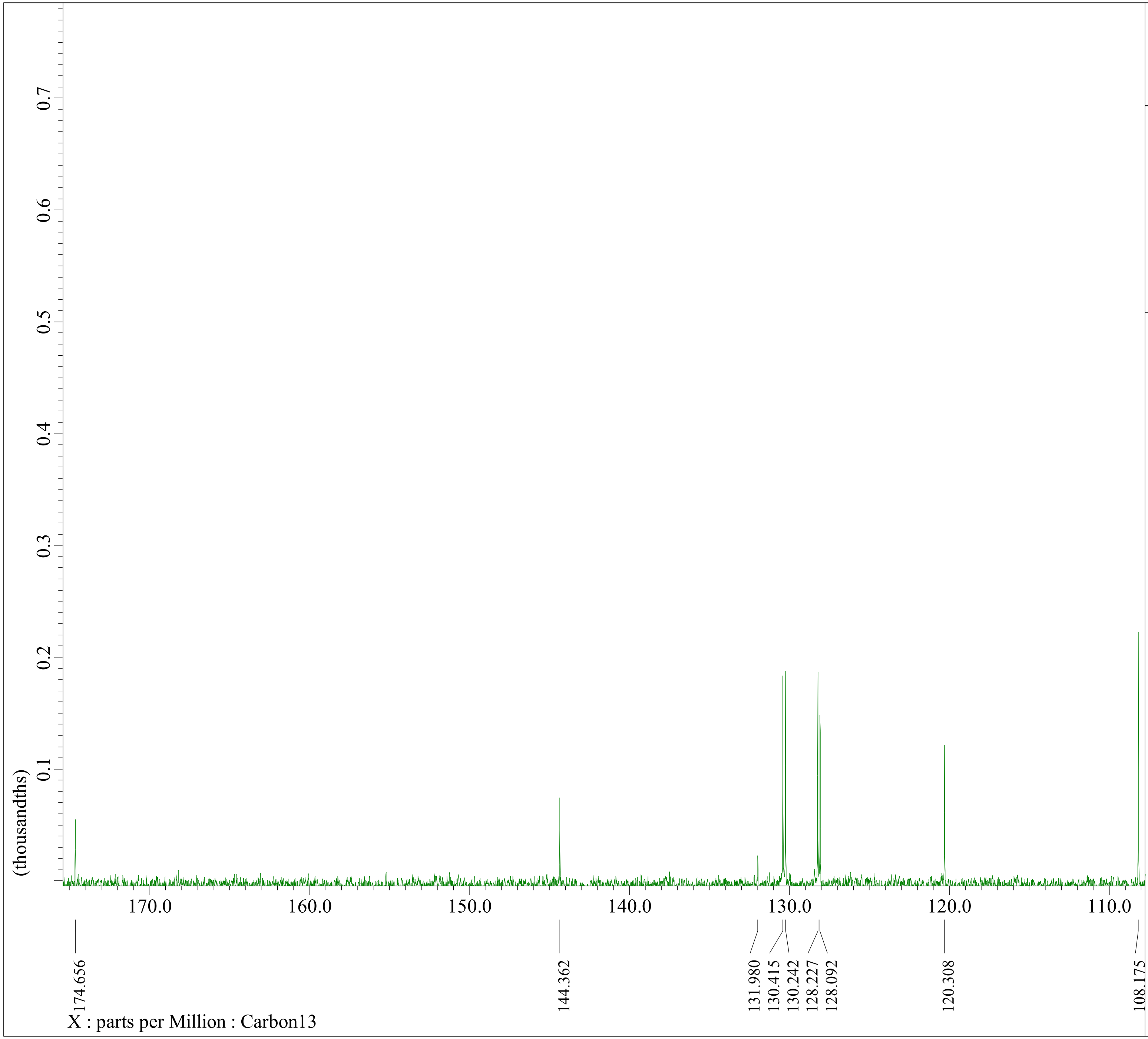

JEOL

---- PROCESSING PARAMETERS ----
sexp(2.0[Hz], 0.0[s])
trapezoid(0[%], 0[%], 80[%], 100[%])
zerofill(1)
fft(1, TRUE, TRUE)
machinephase
ppm

Filename	= PYR-ML-CH3OH-PYR 2
Author	= delta
Experiment	= carbon.jxp
Sample_Id	= PYR-ML-CH3OH-PYR 2
Solvent	= CHLOROFORM-D
Actual_Start_Time	= 18-JUN-2019 09:34:
Revision_Time	= 18-JUN-2019 14:58:
Comment	= single pulse decou
Data_Format	= 1D COMPLEX
Dim_Size	= 26214
X_Domain	= Carbon13
Dim_Title	= Carbon13
Dim_Units	= [ppm]
Dimensions	= X
Spectrometer	= JNM-ECZ500R/S1
Field_Strength	= 11.7473579[T] (500
X_Acq_Duration	= 0.82837504[s]
X_Domain	= Carbon13
X_Freq	= 125.76529768[MHz]
X_Offset	= 100[ppm]
X_Points	= 32768
X_Prescans	= 4
X_Resolution	= 1.20718268[Hz]
X_Sweep	= 39.55696203[kHz]
X_Sweep_Clipped	= 31.64556962[kHz]
Irr_Domain	= Proton
Irr_Freq	= 500.15991521[MHz]
Irr_Offset	= 5.0[ppm]
Blanking	= 2[us]
Clipped	= FALSE
Scans	= 5718
Total_Scans	= 5718
Relaxation_Delay	= 2[s]
Recvr_Gain	= 36
Temp_Get	= 21.5[dC]
X_90_Width	= 11.3[us]
X_Acq_Time	= 0.82837504[s]
X_Angle	= 30[deg]
X_Atn	= 11[dB]
X_Pulse	= 3.76666667[us]
Irr_Atn_Dec	= 30.11[dB]
Irr_Atn_Dec_Calc	= 30.11[dB]
Irr_Atn_Dec_Default_Calc	= 30.11[dB]
Irr_Atn_Noe	= 30.11[dB]
Irr_Dec_Bandwidth_Hz	= 5.97826087[kHz]

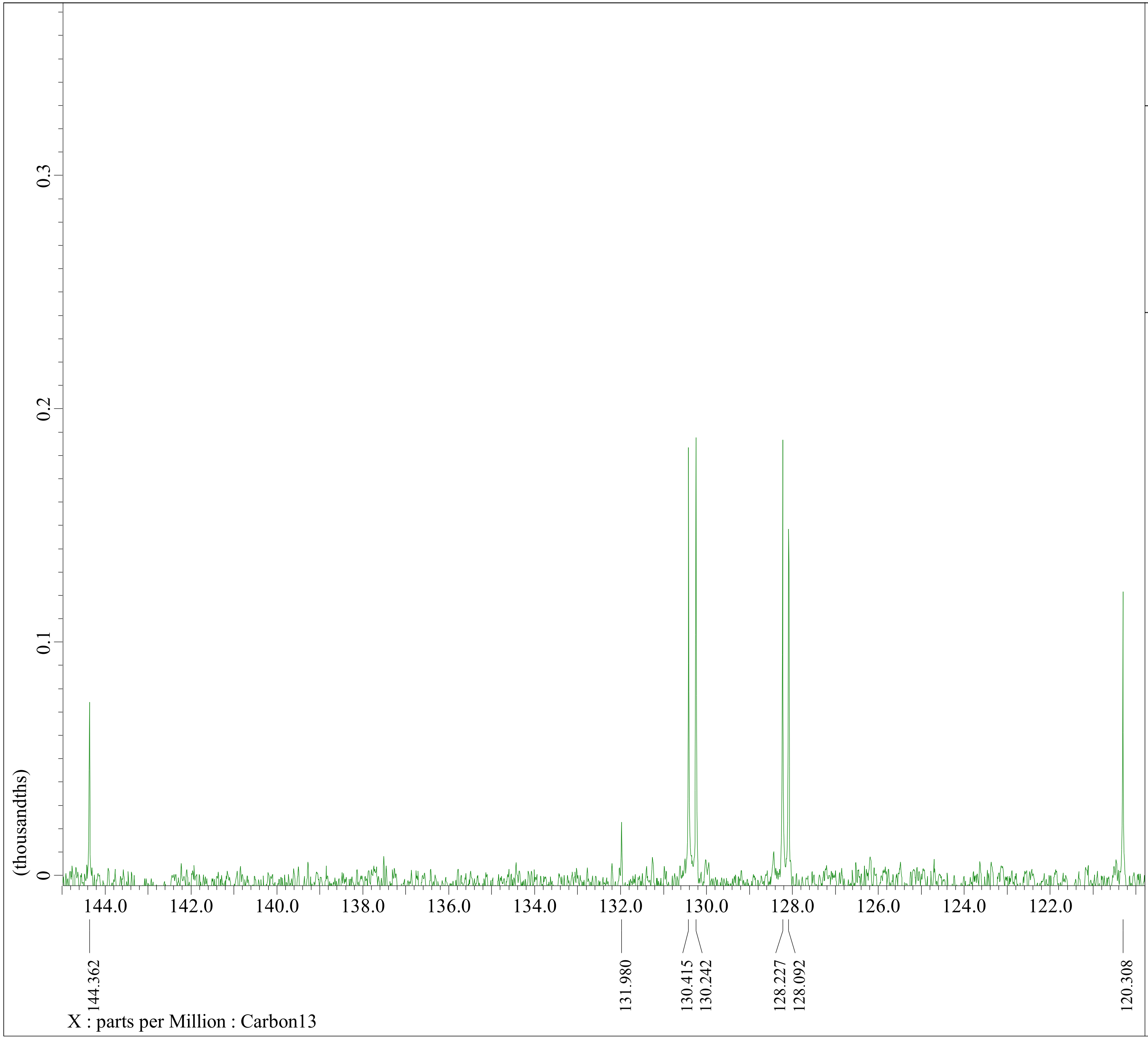

JEOL

```
---- PROCESSING PARAMETERS ----
sexp( 2.0[Hz], 0.0[s] )
trapezoid( 0[%], 0[%], 80[%], 100[%] )
zerofill( 1 )
fft( 1, TRUE, TRUE )
machinephase
ppm
```

```
Filename                  = PYR-ML-CH3OH-PYR 2
Author                    = delta
Experiment                = carbon.jxp
Sample_Id                 = PYR-ML-CH3OH-PYR 2
Solvent                   = CHLOROFORM-D
Actual_Start_Time         = 18-JUN-2019 09:34:
Revision_Time             = 18-JUN-2019 14:58:

Comment                   = single pulse decou
Data_Format               = 1D COMPLEX
Dim_Size                  = 26214
X_Domain                  = Carbon13
Dim_Title                 = Carbon13
Dim_Units                 = [ppm]
Dimensions                = X
Spectrometer              = JNM-ECZ500R/S1

Field_Strength            = 11.7473579[T] (500
X_Acq_Duration            = 0.82837504[s]
X_Domain                  = Carbon13
X_Freq                    = 125.76529768[MHz]
X_Offset                  = 100[ppm]
X_Points                  = 32768
X_Prescans                = 4
X_Resolution              = 1.20718268[Hz]
X_Sweep                   = 39.55696203[kHz]
X_Sweep_Clipped           = 31.64556962[kHz]
Irr_Domain                = Proton
Irr_Freq                  = 500.15991521[MHz]
Irr_Offset                = 5.0[ppm]
Blanking                  = 2[us]
Clipped                   = FALSE
Scans                     = 5718
Total_Scans               = 5718

Relaxation_Delay          = 2[s]
Recvr_Gain                = 36
Temp_Get                  = 21.5[dC]
X_90_Width                = 11.3[us]
X_Acq_Time                = 0.82837504[s]
X_Angle                   = 30[deg]
X_Atn                     = 11[dB]
X_Pulse                   = 3.76666667[us]
Irr_Atn_Dec               = 30.11[dB]
Irr_Atn_Dec_Calc          = 30.11[dB]
Irr_Atn_Dec_Default_Calc  = 30.11[dB]
Irr_Atn_Noe               = 30.11[dB]
Irr_Dec_Bandwidth_Hz      = 5.97826087[kHz]
```

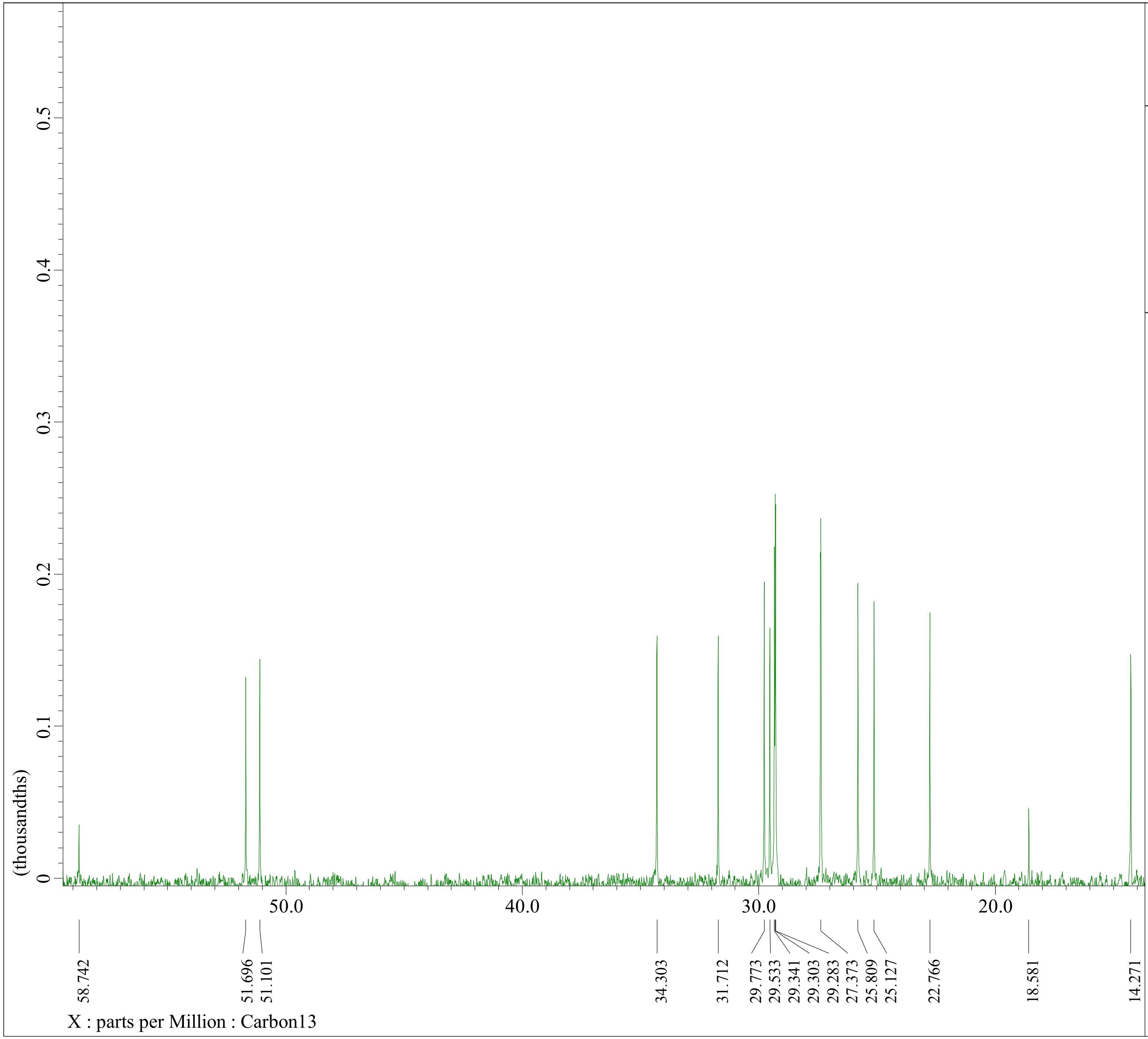


```
---- PROCESSING PARAMETERS ----
sexp( 2.0[Hz], 0.0[s] )
trapezoid( 0[%], 0[%], 80[%], 100[%] )
zerofill( 1 )
fft( 1, TRUE, TRUE )
machinephase
ppm
```

```
Filename                    = PYR-ML-CH3OH-PYR 2
Author                      = delta
Experiment                  = carbon.jxp
Sample_Id                   = PYR-ML-CH3OH-PYR 2
Solvent                     = CHLOROFORM-D
Actual_Start_Time           = 18-JUN-2019 09:34:
Revision_Time               = 18-JUN-2019 14:58:

Comment                     = single pulse decou
Data_Format                 = 1D COMPLEX
Dim_Size                    = 26214
X_Domain                    = Carbon13
Dim_Title                   = Carbon13
Dim_Units                   = [ppm]
Dimensions                  = X
Spectrometer                = JNM-ECZ500R/S1

Field_Strength              = 11.7473579[T] (500
X_Acq_Duration              = 0.82837504[s]
X_Domain                    = Carbon13
X_Freq                      = 125.76529768[MHz]
X_Offset                    = 100[ppm]
X_Points                    = 32768
X_Prescans                  = 4
X_Resolution                = 1.20718268[Hz]
X_Sweep                     = 39.55696203[kHz]
X_Sweep_Clipped             = 31.64556962[kHz]
Irr_Domain                  = Proton
Irr_Freq                    = 500.15991521[MHz]
Irr_Offset                  = 5.0[ppm]
Blanking                    = 2[us]
Clipped                     = FALSE
Scans                       = 5718
Total_Scans                 = 5718

Relaxation_Delay            = 2[s]
Recvr_Gain                  = 36
Temp_Get                    = 21.5[dC]
X_90_Width                  = 11.3[us]
X_Acq_Time                  = 0.82837504[s]
X_Angle                     = 30[deg]
X_Atn                       = 11[dB]
X_Pulse                     = 3.76666667[us]
Irr_Atn_Dec                 = 30.11[dB]
Irr_Atn_Dec_Calc            = 30.11[dB]
Irr_Atn_Dec_Default_Calc    = 30.11[dB]
Irr_Atn_Noe                 = 30.11[dB]
Irr_Dec_Bandwidth_Hz        = 5.97826087[kHz]
```

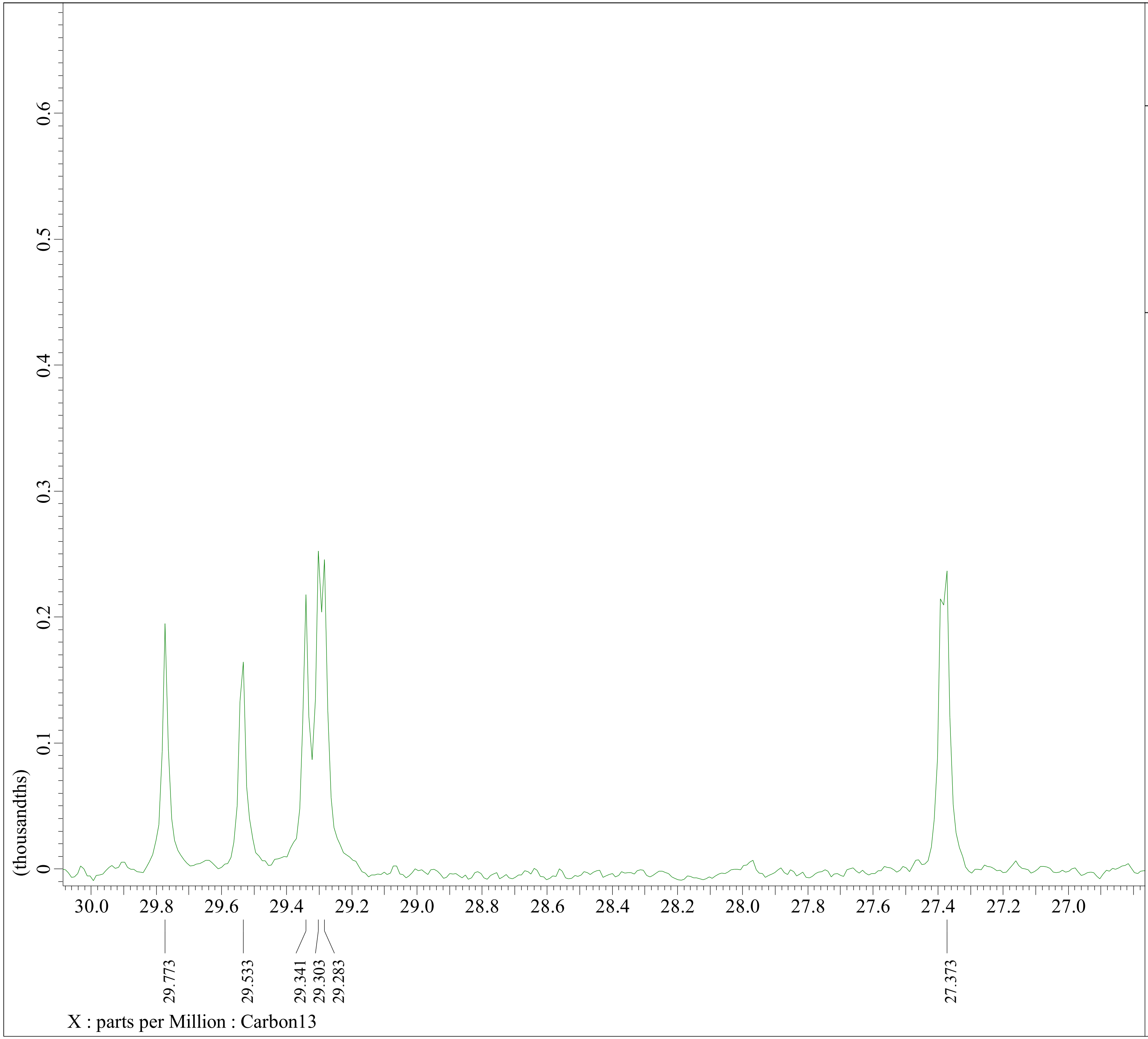


```
---- PROCESSING PARAMETERS ----
sexp( 2.0[Hz], 0.0[s] )
trapezoid( 0[%], 0[%], 80[%], 100[%] )
zerofill( 1 )
fft( 1, TRUE, TRUE )
machinephase
ppm
```

```
Filename                      = PYR-ML-CH3OH-PYR 2
Author                        = delta
Experiment                    = carbon.jxp
Sample_Id                     = PYR-ML-CH3OH-PYR 2
Solvent                       = CHLOROFORM-D
Actual_Start_Time             = 18-JUN-2019 09:34:
Revision_Time                 = 18-JUN-2019 15:14:

Comment                       = single pulse decou
Data_Format                   = 1D COMPLEX
Dim_Size                      = 26214
X_Domain                      = Carbon13
Dim_Title                     = Carbon13
Dim_Units                     = [ppm]
Dimensions                    = X
Spectrometer                  = JNM-ECZ500R/S1

Field_Strength                = 11.7473579[T] (500
X_Acq_Duration                = 0.82837504[s]
X_Domain                      = Carbon13
X_Freq                        = 125.76529768[MHz]
X_Offset                      = 100[ppm]
X_Points                      = 32768
X_Prescans                    = 4
X_Resolution                  = 1.20718268[Hz]
X_Sweep                       = 39.55696203[kHz]
X_Sweep_Clipped               = 31.64556962[kHz]
Irr_Domain                    = Proton
Irr_Freq                      = 500.15991521[MHz]
Irr_Offset                    = 5.0[ppm]
Blanking                      = 2[us]
Clipped                       = FALSE
Scans                         = 5718
Total_Scans                   = 5718

Relaxation_Delay              = 2[s]
Recvr_Gain                    = 36
Temp_Get                      = 21.5[dC]
X_90_Width                    = 11.3[us]
X_Acq_Time                    = 0.82837504[s]
X_Angle                       = 30[deg]
X_Atn                         = 11[dB]
X_Pulse                       = 3.76666667[us]
Irr_Atn_Dec                   = 30.11[dB]
Irr_Atn_Dec_Calc              = 30.11[dB]
Irr_Atn_Dec_Default_Calc      = 30.11[dB]
Irr_Atn_Noe                   = 30.11[dB]
Irr_Dec_Bandwidth_Hz          = 5.97826087[kHz]
```

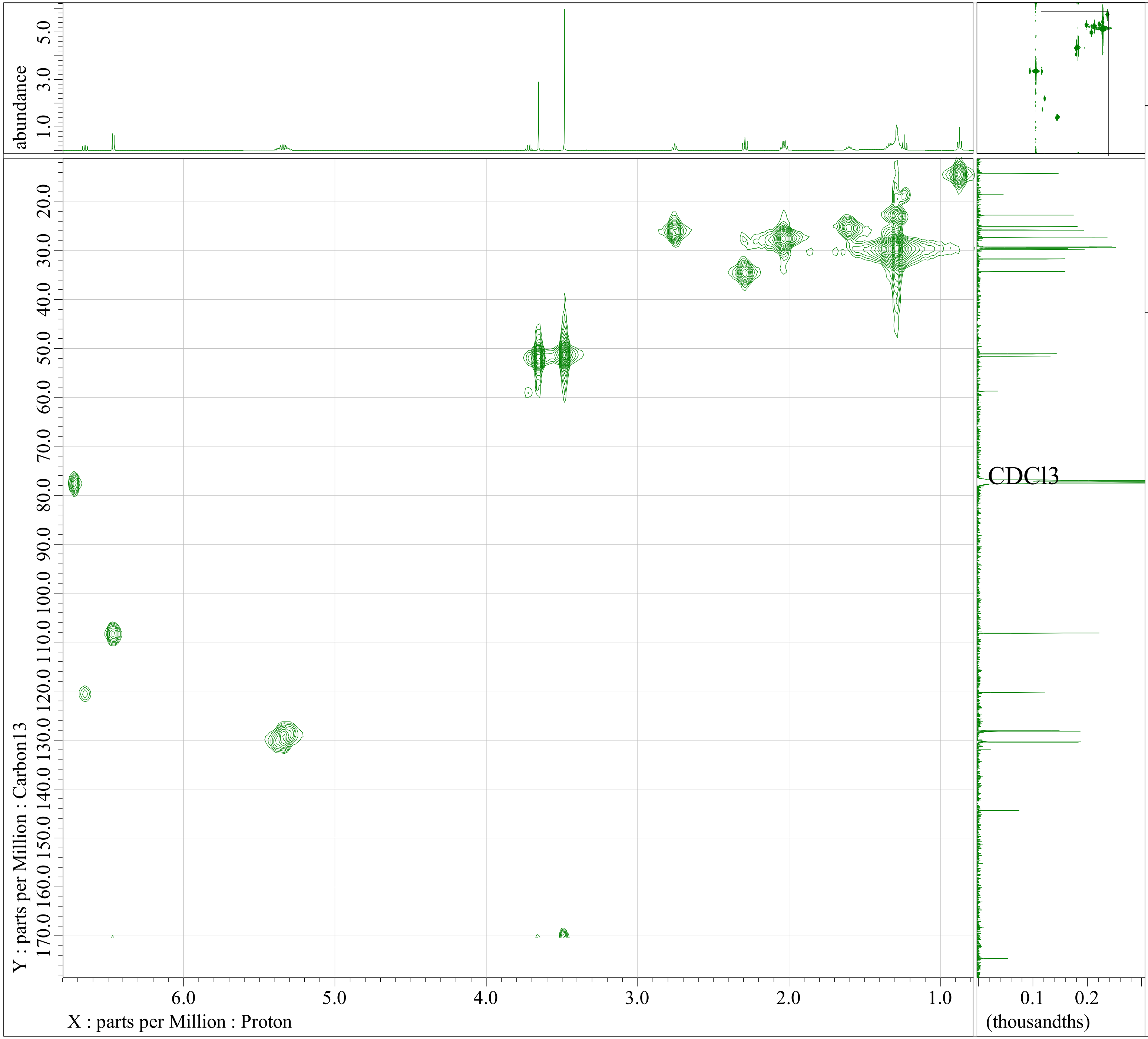


```
---- PROCESSING PARAMETERS ----
sexp( 2.0[Hz], 0.0[s] )
trapezoid( 0[%], 0[%], 80[%], 100[%] )
zerofill( 1 )
fft( 1, TRUE, TRUE )
machinephase
ppm
```

```
Filename             = PYR-ML-CH3OH-PYR 2
Author               = delta
Experiment           = hmqc.jxp
Sample_Id            = PYR-ML-CH3OH-PYR 2
Solvent              = CHLOROFORM-D
Actual_Start_Time    = 18-JUN-2019 14:08:
Revision_Time        = 18-JUN-2019 15:18:

Comment              = gradient enhanced
Data_Format          = 2D REAL REAL
Dim_Size             = 819, 240
X_Domain             = Proton
Y_Domain             = Carbon13
Dim_Title            = Proton Carbon13
Dim_Units            = [ppm] [ppm]
Dimensions           = X Y
Spectrometer         = JNM-ECZ500R/S1

Field_Strength       = 11.7473579[T] (500
X_Acq_Duration       = 0.10911744[s]
X_Domain             = Proton
X_Freq               = 500.15991521[MHz]
X_Offset             = 5.0[ppm]
X_Points             = 1024
X_Prescans           = 4
X_Resolution         = 9.16443788[Hz]
X_Sweep              = 9.38438438[kHz]
X_Sweep_Clipped      = 7.50750751[kHz]
Y_Domain             = Carbon13
Y_Freq               = 125.76529768[MHz]
Y_Offset             = 85[ppm]
Y_Points             = 120
Y_Prescans           = 0
Y_Resolution         = 83.53828058[Hz]
Y_Sweep              = 21.38579983[kHz]
Tri_Domain           = Proton
Tri_Freq             = 500.15991521[MHz]
Tri_Offset           = 5.0[ppm]
Blanking             = 2[us]
Clipped              = FALSE
Scans                = 20
Total_Scans          = 2400

Relaxation_Delay     = 1.5[s]
Recvr_Gain           = 76
Temp_Get             = 21.8[dC]
X_Acq_Time           = 0.10911744[s]
X_Atn                = 8.9[dB]
```

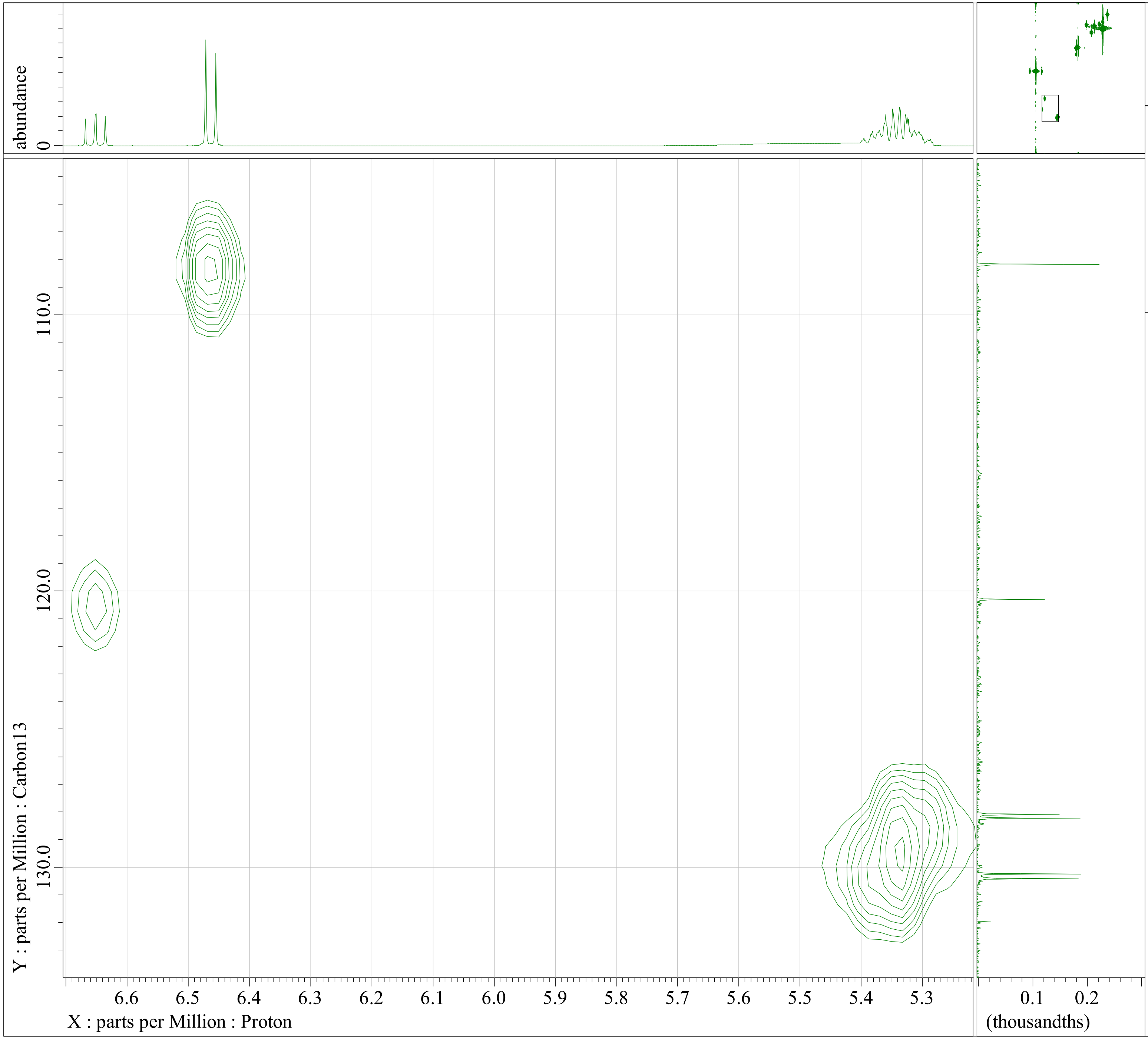


```
---- PROCESSING PARAMETERS ----
sinbell4( -60, 160 )
zerofill( 1 )
fft( 1, TRUE, TRUE )
ppm
[transpose]
sinbell4( -60, 160 )
zerofill( 2 )
fft( 1, TRUE, TRUE )
ppm
abs
[transpose]
```

```
Filename                 = PYR-ML-CH3OH-PYR 2
Author                   = delta
Experiment               = hmqc.jxp
Sample_Id                = PYR-ML-CH3OH-PYR 2
Solvent                  = CHLOROFORM-D
Actual_Start_Time        = 18-JUN-2019 14:08:
Revision_Time            = 18-JUN-2019 15:18:

Comment                  = gradient enhanced
Data_Format              = 2D REAL REAL
Dim_Size                 = 819, 240
X_Domain                 = Proton
Y_Domain                 = Carbon13
Dim_Title                = Proton Carbon13
Dim_Units                = [ppm] [ppm]
Dimensions               = X Y
Spectrometer             = JNM-ECZ500R/S1

Field_Strength           = 11.7473579[T] (500
X_Acq_Duration           = 0.10911744[s]
X_Domain                 = Proton
X_Freq                   = 500.15991521[MHz]
X_Offset                 = 5.0[ppm]
X_Points                 = 1024
X_Prescans               = 4
X_Resolution             = 9.16443788[Hz]
X_Sweep                  = 9.38438438[kHz]
X_Sweep_Clipped          = 7.50750751[kHz]
Y_Domain                 = Carbon13
Y_Freq                   = 125.76529768[MHz]
Y_Offset                 = 85[ppm]
Y_Points                 = 120
Y_Prescans               = 0
Y_Resolution             = 83.53828058[Hz]
Y_Sweep                  = 21.38579983[kHz]
Tri_Domain               = Proton
Tri_Freq                 = 500.15991521[MHz]
Tri_Offset               = 5.0[ppm]
Blanking                 = 2[us]
Clipped                  = FALSE
Scans                    = 20
Total_Scans              = 2400

Relaxation_Delay         = 1.5[s]
Recvr_Gain               = 76
Temp_Get                 = 21.8[dC]
X_Acq_Time               = 0.10911744[s]
X_Atn                    = 8.9[dB]
```

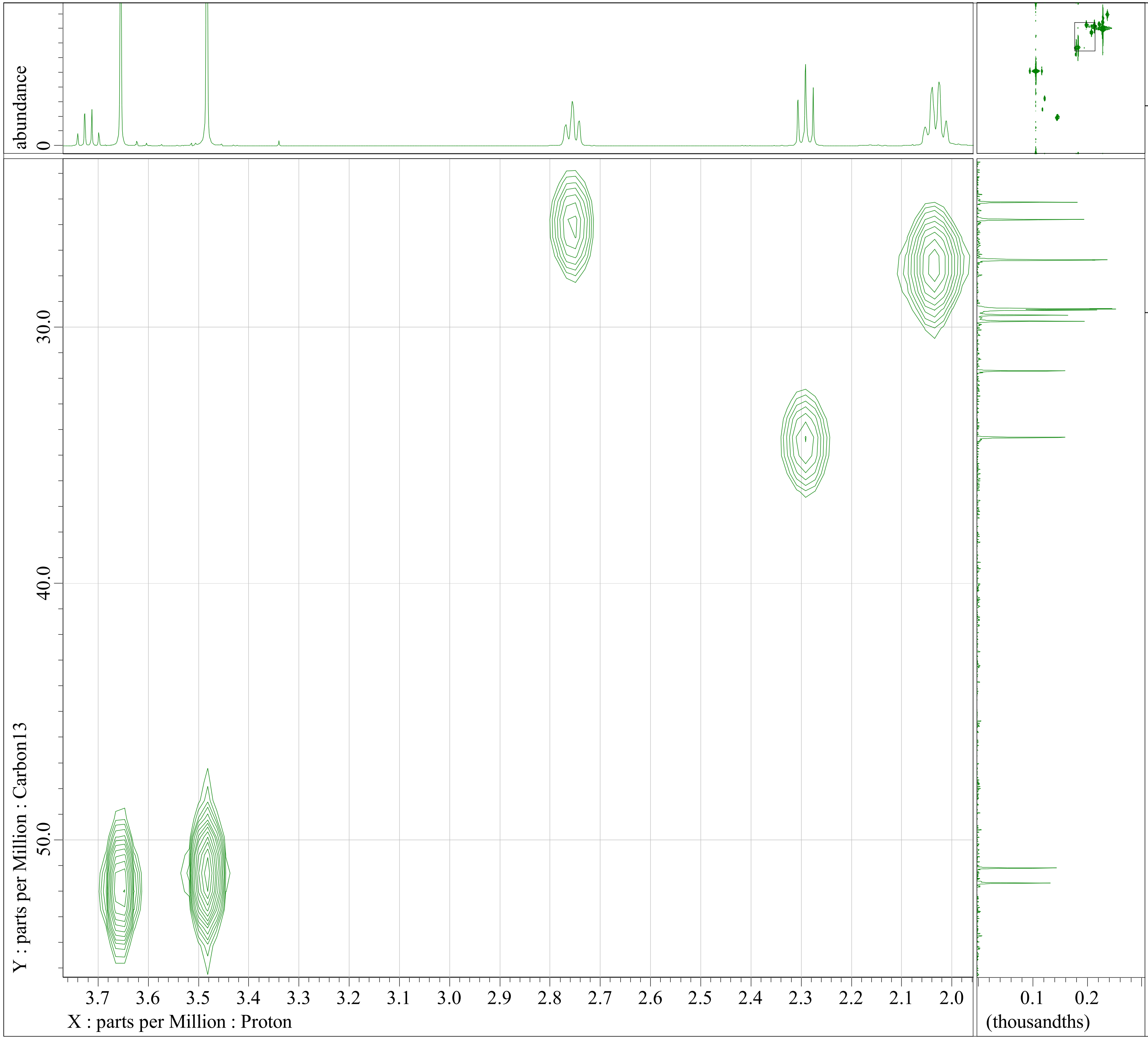

JEOL

```
---- PROCESSING PARAMETERS ----
sinbell4( -60, 160 )
zerofill( 1 )
fft( 1, TRUE, TRUE )
ppm
[transpose]
sinbell4( -60, 160 )
zerofill( 2 )
fft( 1, TRUE, TRUE )
ppm
abs
[transpose]
```

```
Filename              = PYR-ML-CH3OH-PYR 2
Author                = delta
Experiment            = hmqc.jxp
Sample_Id             = PYR-ML-CH3OH-PYR 2
Solvent               = CHLOROFORM-D
Actual_Start_Time     = 18-JUN-2019 14:08:
Revision_Time         = 18-JUN-2019 15:18:

Comment               = gradient enhanced
Data_Format           = 2D REAL REAL
Dim_Size              = 819, 240
X_Domain              = Proton
Y_Domain              = Carbon13
Dim_Title             = Proton Carbon13
Dim_Units             = [ppm] [ppm]
Dimensions            = X Y
Spectrometer          = JNM-ECZ500R/S1

Field_Strength        = 11.7473579[T] (500
X_Acq_Duration        = 0.10911744[s]
X_Domain              = Proton
X_Freq                = 500.15991521[MHz]
X_Offset              = 5.0[ppm]
X_Points              = 1024
X_Prescans            = 4
X_Resolution          = 9.16443788[Hz]
X_Sweep               = 9.38438438[kHz]
X_Sweep_Clipped       = 7.50750751[kHz]
Y_Domain              = Carbon13
Y_Freq                = 125.76529768[MHz]
Y_Offset              = 85[ppm]
Y_Points              = 120
Y_Prescans            = 0
Y_Resolution          = 83.53828058[Hz]
Y_Sweep               = 21.38579983[kHz]
Tri_Domain            = Proton
Tri_Freq              = 500.15991521[MHz]
Tri_Offset            = 5.0[ppm]
Blanking              = 2[us]
Clipped               = FALSE
Scans                 = 20
Total_Scans           = 2400

Relaxation_Delay      = 1.5[s]
Recvr_Gain            = 76
Temp_Get              = 21.8[dC]
X_Acq_Time            = 0.10911744[s]
X_Atn                 = 8.9[dB]
```

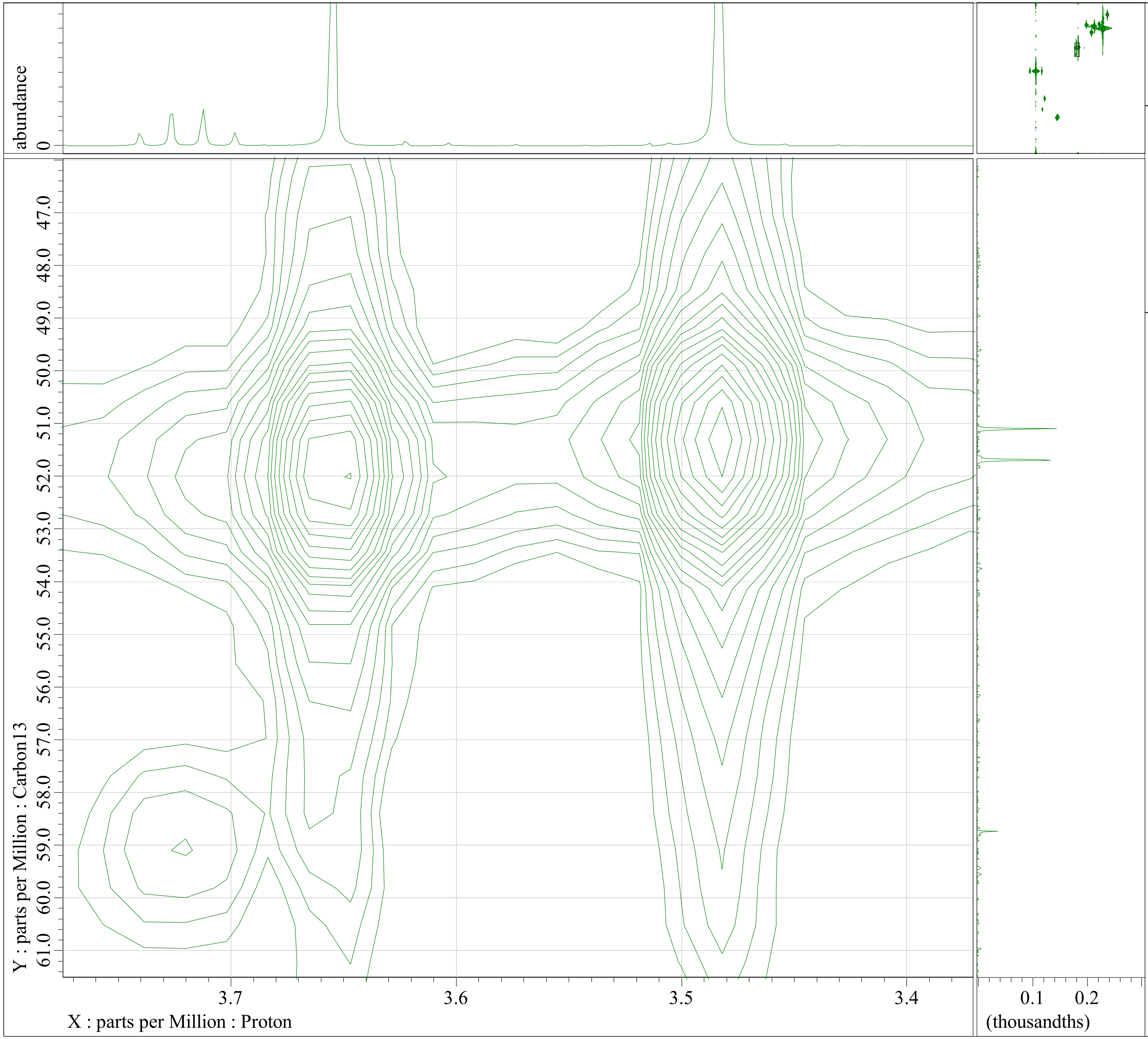

JEOL

---- PROCESSING PARAMETERS ----
sinbell4(-60, 160)
zerofill(1)
fft(1, TRUE, TRUE)
ppm
[transpose]
sinbell4(-60, 160)
zerofill(2)
fft(1, TRUE, TRUE)
ppm
abs
[transpose]

Filename	= PYR-ML-CH3OH-PYR 2
Author	= delta
Experiment	= hmqc.jxp
Sample_Id	= PYR-ML-CH3OH-PYR 2
Solvent	= CHLOROFORM-D
Actual_Start_Time	= 18-JUN-2019 14:08:
Revision_Time	= 18-JUN-2019 15:18:
Comment	= gradient enhanced
Data_Format	= 2D REAL REAL
Dim_Size	= 819, 240
X_Domain	= Proton
Y_Domain	= Carbon13
Dim_Title	= Proton Carbon13
Dim_Units	= [ppm] [ppm]
Dimensions	= X Y
Spectrometer	= JNM-ECZ500R/S1
Field_Strength	= 11.7473579[T] (500
X_Acq_Duration	= 0.10911744[s]
X_Domain	= Proton
X_Freq	= 500.15991521[MHz]
X_Offset	= 5.0[ppm]
X_Points	= 1024
X_Prescans	= 4
X_Resolution	= 9.16443788[Hz]
X_Sweep	= 9.38438438[kHz]
X_Sweep_Clipped	= 7.50750751[kHz]
Y_Domain	= Carbon13
Y_Freq	= 125.76529768[MHz]
Y_Offset	= 85[ppm]
Y_Points	= 120
Y_Prescans	= 0
Y_Resolution	= 83.53828058[Hz]
Y_Sweep	= 21.38579983[kHz]
Tri_Domain	= Proton
Tri_Freq	= 500.15991521[MHz]
Tri_Offset	= 5.0[ppm]
Blanking	= 2[us]
Clipped	= FALSE
Scans	= 20
Total_Scans	= 2400
Relaxation_Delay	= 1.5[s]
Recvr_Gain	= 76
Temp_Get	= 21.8[dC]
X_Acq_Time	= 0.10911744[s]
X_Atn	= 8.9[dB]

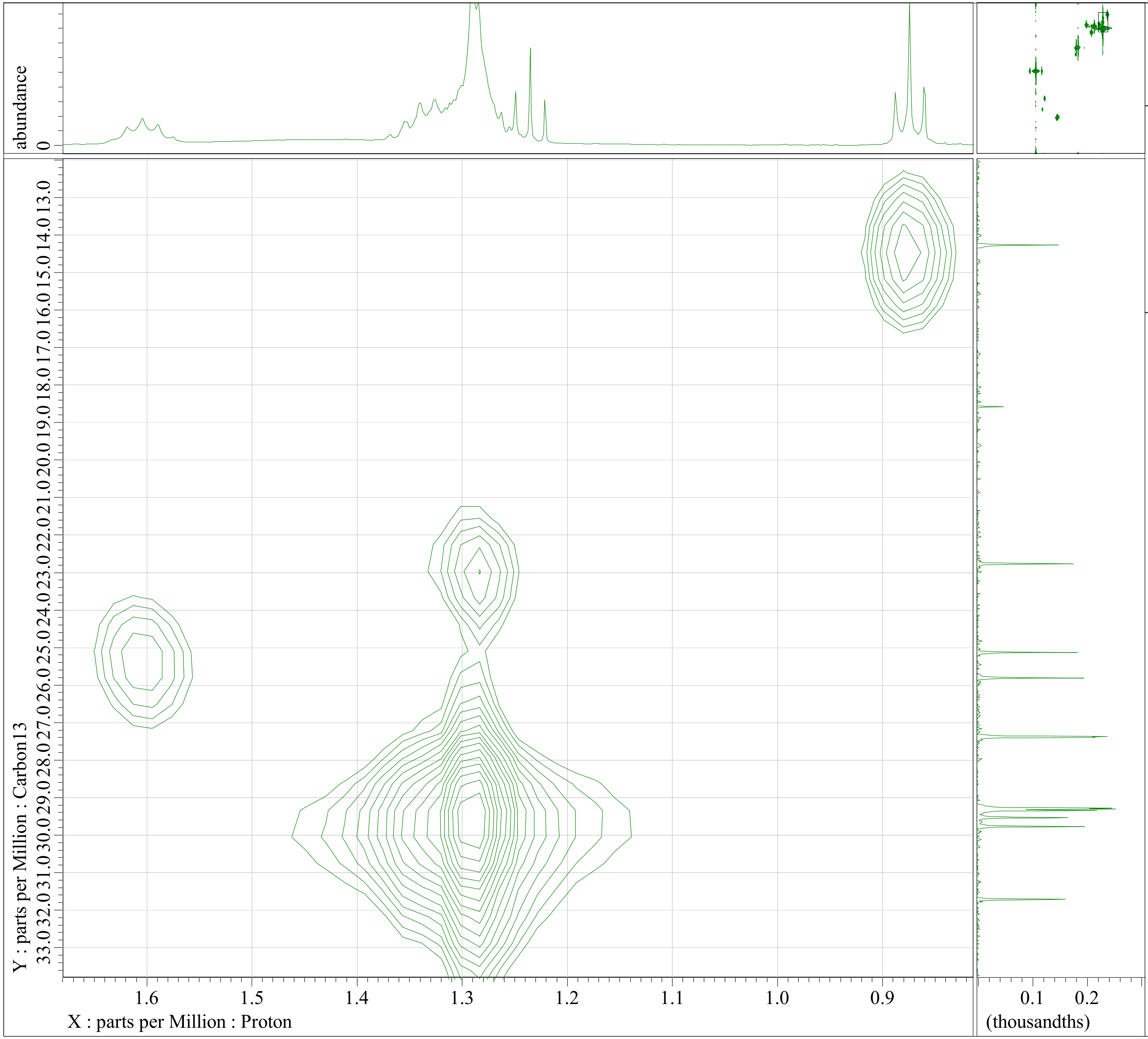

JEOL

```
---- PROCESSING PARAMETERS ----
sinbell4( -60, 160 )
zerofill( 1 )
fft( 1, TRUE, TRUE )
ppm
[transpose]
sinbell4( -60, 160 )
zerofill( 2 )
fft( 1, TRUE, TRUE )
ppm
abs
[transpose]
```

```
Filename             = PYR-ML-CH3OH-PYR 2
Author               = delta
Experiment           = hmqc.jxp
Sample_Id            = PYR-ML-CH3OH-PYR 2
Solvent              = CHLOROFORM-D
Actual_Start_Time    = 18-JUN-2019 14:08:
Revision_Time        = 18-JUN-2019 15:18:

Comment              = gradient enhanced
Data_Format          = 2D REAL REAL
Dim_Size             = 819, 240
X_Domain             = Proton
Y_Domain             = Carbon13
Dim_Title            = Proton Carbon13
Dim_Units            = [ppm] [ppm]
Dimensions           = X Y
Spectrometer         = JNM-ECZ500R/S1

Field_Strength       = 11.7473579[T] (500
X_Acq_Duration       = 0.10911744[s]
X_Domain             = Proton
X_Freq               = 500.15991521[MHz]
X_Offset             = 5.0[ppm]
X_Points             = 1024
X_Prescans           = 4
X_Resolution         = 9.16443788[Hz]
X_Sweep              = 9.38438438[kHz]
X_Sweep_Clipped      = 7.50750751[kHz]
Y_Domain             = Carbon13
Y_Freq               = 125.76529768[MHz]
Y_Offset             = 85[ppm]
Y_Points             = 120
Y_Prescans           = 0
Y_Resolution         = 83.53828058[Hz]
Y_Sweep              = 21.38579983[kHz]
Tri_Domain           = Proton
Tri_Freq             = 500.15991521[MHz]
Tri_Offset           = 5.0[ppm]
Blanking             = 2[us]
Clipped              = FALSE
Scans                = 20
Total_Scans          = 2400

Relaxation_Delay     = 1.5[s]
Recvr_Gain           = 76
Temp_Get             = 21.8[dC]
X_Acq_Time           = 0.10911744[s]
X_Atn                = 8.9[dB]
```

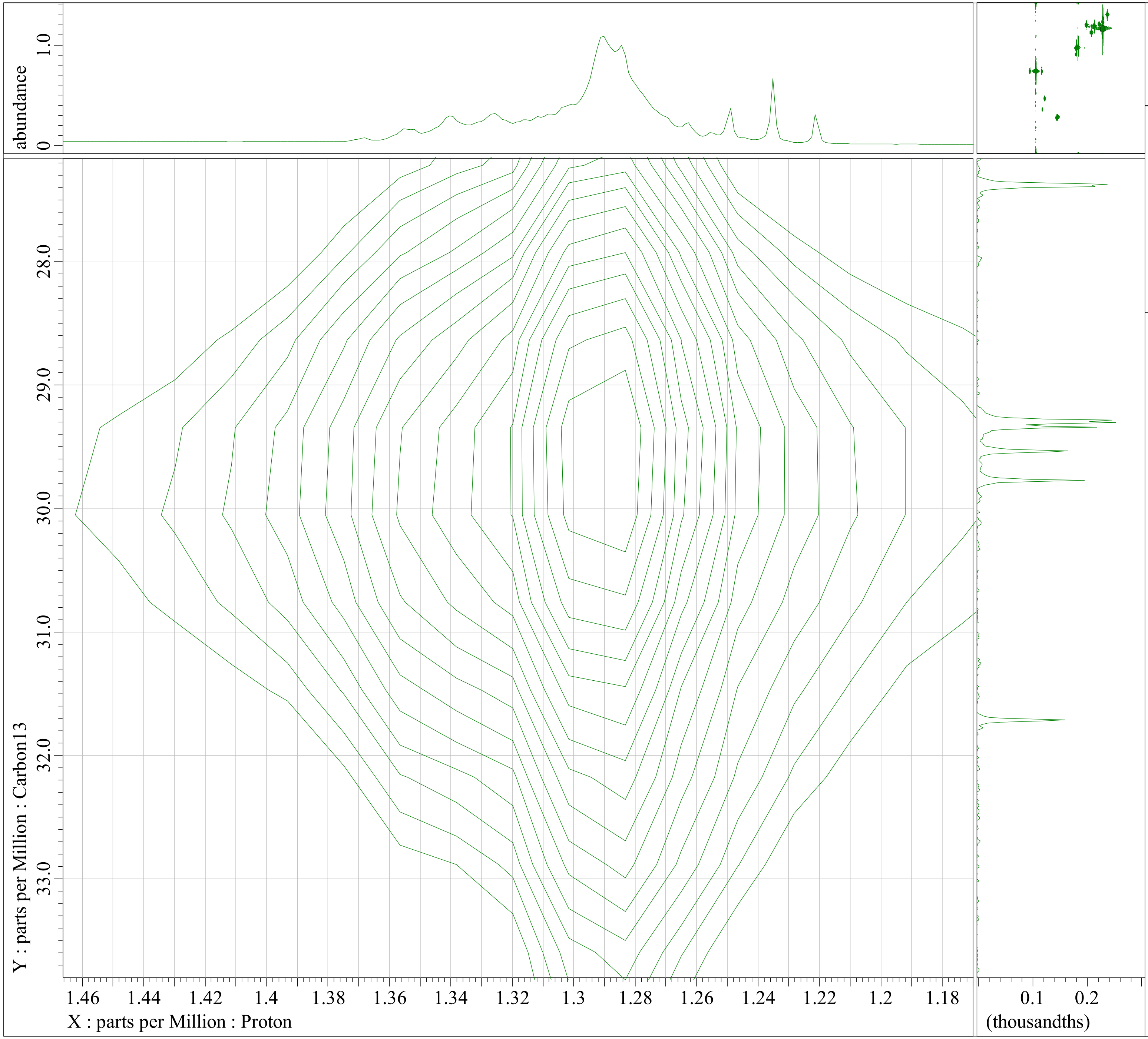

JEOL

---- PROCESSING PARAMETERS ----
sexp(0.2[Hz], 0.0[s])
trapezoid(0[%], 0[%], 80[%], 100[%])
zerofill(1)
fft(1, TRUE, TRUE)
machinephase
ppm

Filename	= PYR-ML-CH3OH-PYR 2
Author	= delta
Experiment	= hmqc.jxp
Sample_Id	= PYR-ML-CH3OH-PYR 2
Solvent	= CHLOROFORM-D
Actual_Start_Time	= 18-JUN-2019 14:08:
Revision_Time	= 18-JUN-2019 15:18:
Comment	= gradient enhanced
Data_Format	= 2D REAL REAL
Dim_Size	= 819, 240
X_Domain	= Proton
Y_Domain	= Carbon13
Dim_Title	= Proton Carbon13
Dim_Units	= [ppm] [ppm]
Dimensions	= X Y
Spectrometer	= JNM-ECZ500R/S1
Field_Strength	= 11.7473579[T] (500
X_Acq_Duration	= 0.10911744[s]
X_Domain	= Proton
X_Freq	= 500.15991521[MHz]
X_Offset	= 5.0[ppm]
X_Points	= 1024
X_Prescans	= 4
X_Resolution	= 9.16443788[Hz]
X_Sweep	= 9.38438438[kHz]
X_Sweep_Clipped	= 7.50750751[kHz]
Y_Domain	= Carbon13
Y_Freq	= 125.76529768[MHz]
Y_Offset	= 85[ppm]
Y_Points	= 120
Y_Prescans	= 0
Y_Resolution	= 83.53828058[Hz]
Y_Sweep	= 21.38579983[kHz]
Tri_Domain	= Proton
Tri_Freq	= 500.15991521[MHz]
Tri_Offset	= 5.0[ppm]
Blanking	= 2[us]
Clipped	= FALSE
Scans	= 20
Total_Scans	= 2400
Relaxation_Delay	= 1.5[s]
Recvr_Gain	= 76
Temp_Get	= 21.8[dC]
X_Acq_Time	= 0.10911744[s]
X_Atn	= 8.9[dB]

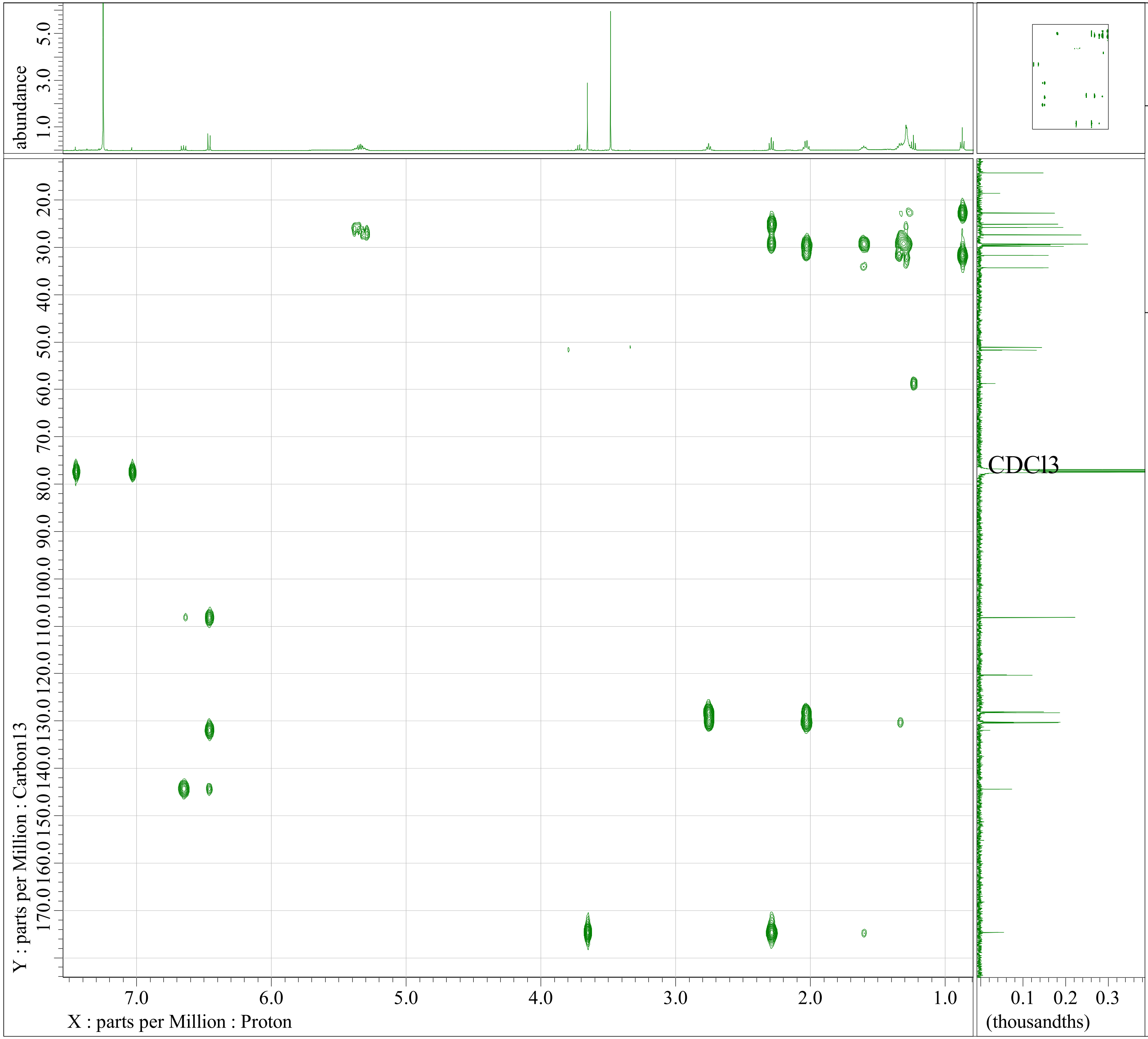

JEOL

---- PROCESSING PARAMETERS ----
gauss(5.0[Hz], 0.0[s])
sinbell_auto
zerofill(1)
fft(1, TRUE, TRUE)
ppm
[transpose]
sinbell4(-60, 160)
trapezoid(0[%], 5[%], 80[%], 100[%])
zerofill(2)
fft(1, TRUE, TRUE)
ppm
abs

Filename = PYR-ML-CH3OH-PYR 2-U_
Author = delta
Experiment = hmbc.jxp
Sample_Id = PYR-ML-CH3OH-PYR 2-U
Solvent = CHLOROFORM-D
Actual_Start_Time = 18-JUN-2019 15:15:44
Revision_Time = 18-JUN-2019 16:26:40

Comment = gradient enhanced HMB
Data_Format = 2D REAL REAL
Dim_Size = 1638, 512
X_Domain = Proton
Y_Domain = Carbon13
Dim_Title = Proton Carbon13
Dim_Units = [ppm] [ppm]
Dimensions = X Y
Spectrometer = JNM-ECZ500R/S1

Field_Strength = 11.7473579[T] (500[MH
X_Acq_Duration = 0.21823488[s]
X_Domain = Proton
X_Freq = 500.15991521[MHz]
X_Offset = 5.0[ppm]
X_Points = 2048
X_Prescans = 4
X_Resolution = 4.58221894[Hz]
X_Sweep = 9.38438438[kHz]
X_Sweep_Clipped = 7.50750751[kHz]
Y_Domain = Carbon13
Y_Freq = 125.76529768[MHz]
Y_Offset = 100[ppm]
Y_Points = 256
Y_Prescans = 0
Y_Resolution = 122.83805031[Hz]
Y_Sweep = 31.44654088[kHz]
Tri_Domain = Proton
Tri_Freq = 500.15991521[MHz]
Tri_Offset = 5.0[ppm]
Blanking = 2[us]
Clipped = FALSE
Scans = 8
Total_Scans = 2048

Relaxation_Delay = 1.5[s]
Recvr_Gain = 76
Temp_Get = 21.7[dC]
X_Acq_Time = 0.21823488[s]
X_Atn = 8.9[dB]

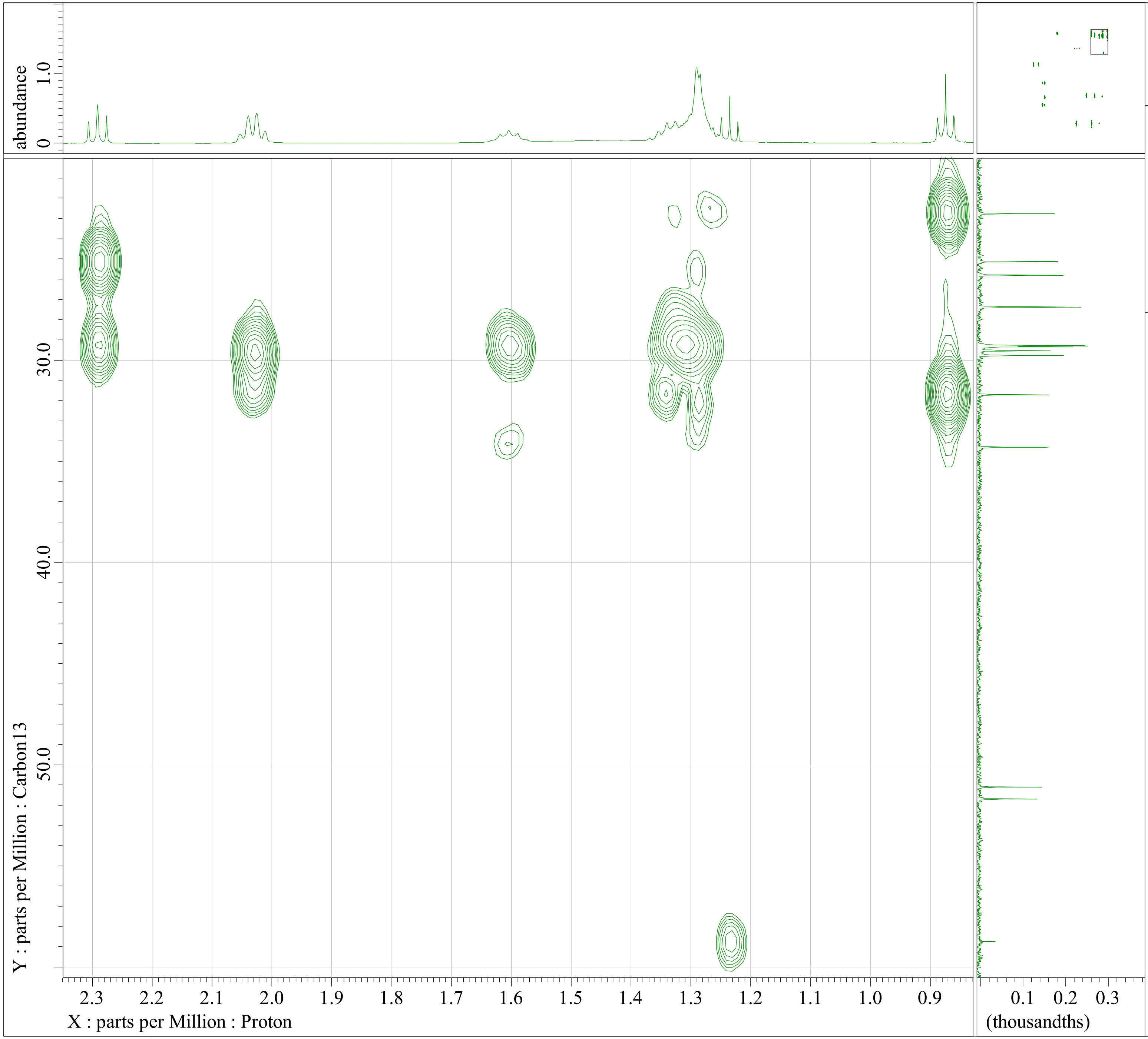

---- PROCESSING PARAMETERS ----
sexp(0.2[Hz], 0.0[s])
trapezoid(0[%], 0[%], 80[%], 100[%])
zerofill(1)
fft(1, TRUE, TRUE)
machinephase
ppm

Filename	= PYR-ML-CH3OH-PYR 2-U_
Author	= delta
Experiment	= hmbc.jxp
Sample_Id	= PYR-ML-CH3OH-PYR 2-U
Solvent	= CHLOROFORM-D
Actual_Start_Time	= 18-JUN-2019 15:15:44
Revision_Time	= 18-JUN-2019 16:26:40
Comment	= gradient enhanced HMB
Data_Format	= 2D REAL REAL
Dim_Size	= 1638, 512
X_Domain	= Proton
Y_Domain	= Carbon13
Dim_Title	= Proton Carbon13
Dim_Units	= [ppm] [ppm]
Dimensions	= X Y
Spectrometer	= JNM-ECZ500R/S1
Field_Strength	= 11.7473579[T] (500[MH
X_Acq_Duration	= 0.21823488[s]
X_Domain	= Proton
X_Freq	= 500.15991521[MHz]
X_Offset	= 5.0[ppm]
X_Points	= 2048
X_Prescans	= 4
X_Resolution	= 4.58221894[Hz]
X_Sweep	= 9.38438438[kHz]
X_Sweep_Clipped	= 7.50750751[kHz]
Y_Domain	= Carbon13
Y_Freq	= 125.76529768[MHz]
Y_Offset	= 100[ppm]
Y_Points	= 256
Y_Prescans	= 0
Y_Resolution	= 122.83805031[Hz]
Y_Sweep	= 31.44654088[kHz]
Tri_Domain	= Proton
Tri_Freq	= 500.15991521[MHz]
Tri_Offset	= 5.0[ppm]
Blanking	= 2[us]
Clipped	= FALSE
Scans	= 8
Total_Scans	= 2048
Relaxation_Delay	= 1.5[s]
Recvr_Gain	= 76
Temp_Get	= 21.7[dC]
X_Acq_Time	= 0.21823488[s]
X_Atn	= 8.9[dB]

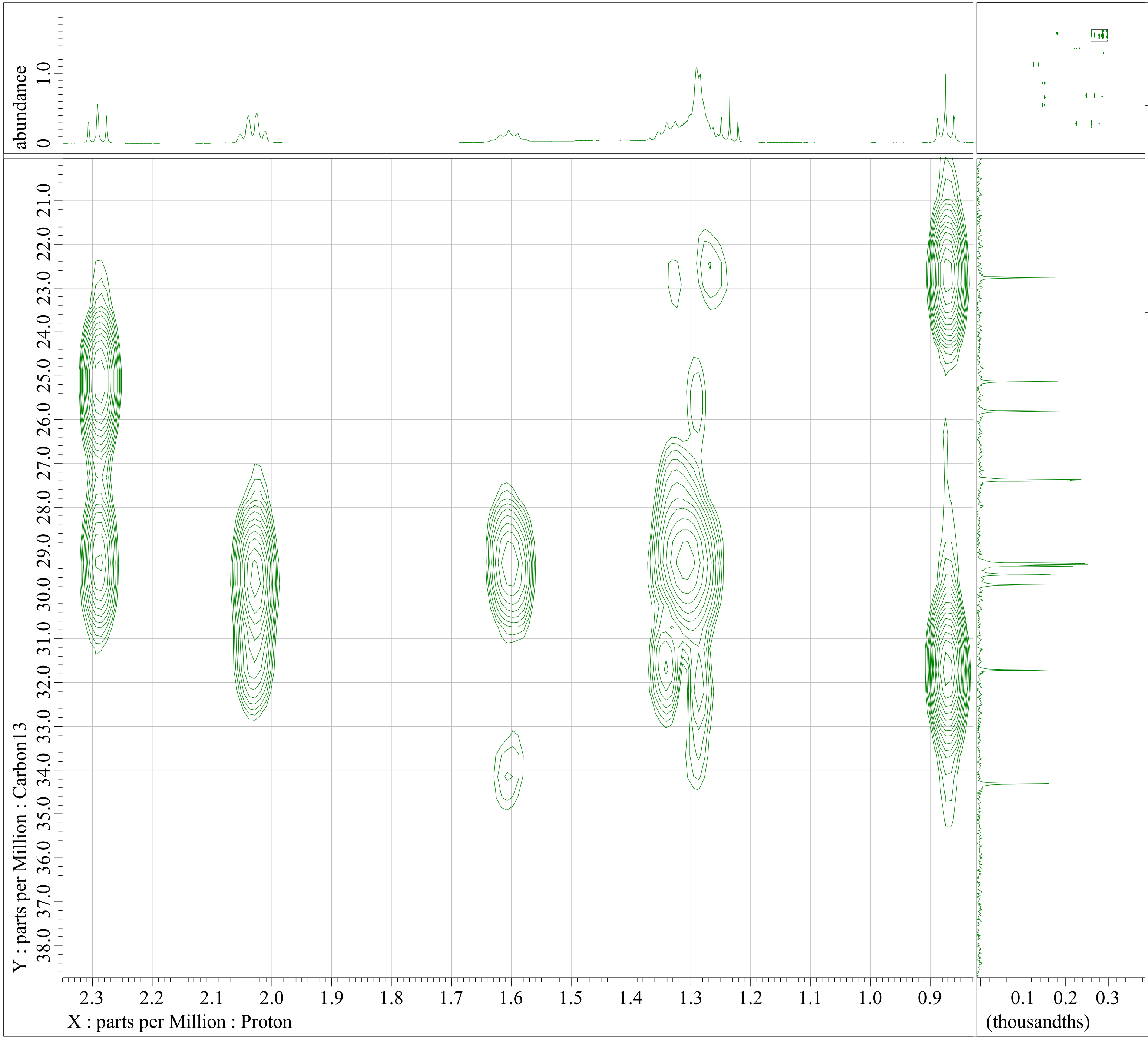

JEOL

---- PROCESSING PARAMETERS ----
gauss(5.0[Hz], 0.0[s])
sinbell_auto
zerofill(1)
fft(1, TRUE, TRUE)
ppm
[transpose]
sinbell4(-60, 160)
trapezoid(0[%], 5[%], 80[%], 100[%])
zerofill(2)
fft(1, TRUE, TRUE)
ppm
abs

Filename	= PYR-ML-CH3OH-PYR 2-U_
Author	= delta
Experiment	= hmbc.jxp
Sample_Id	= PYR-ML-CH3OH-PYR 2-U
Solvent	= CHLOROFORM-D
Actual_Start_Time	= 18-JUN-2019 15:15:44
Revision_Time	= 18-JUN-2019 16:26:40
Comment	= gradient enhanced HMB
Data_Format	= 2D REAL REAL
Dim_Size	= 1638, 512
X_Domain	= Proton
Y_Domain	= Carbon13
Dim_Title	= Proton Carbon13
Dim_Units	= [ppm] [ppm]
Dimensions	= X Y
Spectrometer	= JNM-ECZ500R/S1
Field_Strength	= 11.7473579[T] (500[MH
X_Acq_Duration	= 0.21823488[s]
X_Domain	= Proton
X_Freq	= 500.15991521[MHz]
X_Offset	= 5.0[ppm]
X_Points	= 2048
X_Prescans	= 4
X_Resolution	= 4.58221894[Hz]
X_Sweep	= 9.38438438[kHz]
X_Sweep_Clipped	= 7.50750751[kHz]
Y_Domain	= Carbon13
Y_Freq	= 125.76529768[MHz]
Y_Offset	= 100[ppm]
Y_Points	= 256
Y_Prescans	= 0
Y_Resolution	= 122.83805031[Hz]
Y_Sweep	= 31.44654088[kHz]
Tri_Domain	= Proton
Tri_Freq	= 500.15991521[MHz]
Tri_Offset	= 5.0[ppm]
Blanking	= 2[us]
Clipped	= FALSE
Scans	= 8
Total_Scans	= 2048
Relaxation_Delay	= 1.5[s]
Recvr_Gain	= 76
Temp_Get	= 21.7[dC]
X_Acq_Time	= 0.21823488[s]
X_Atn	= 8.9[dB]

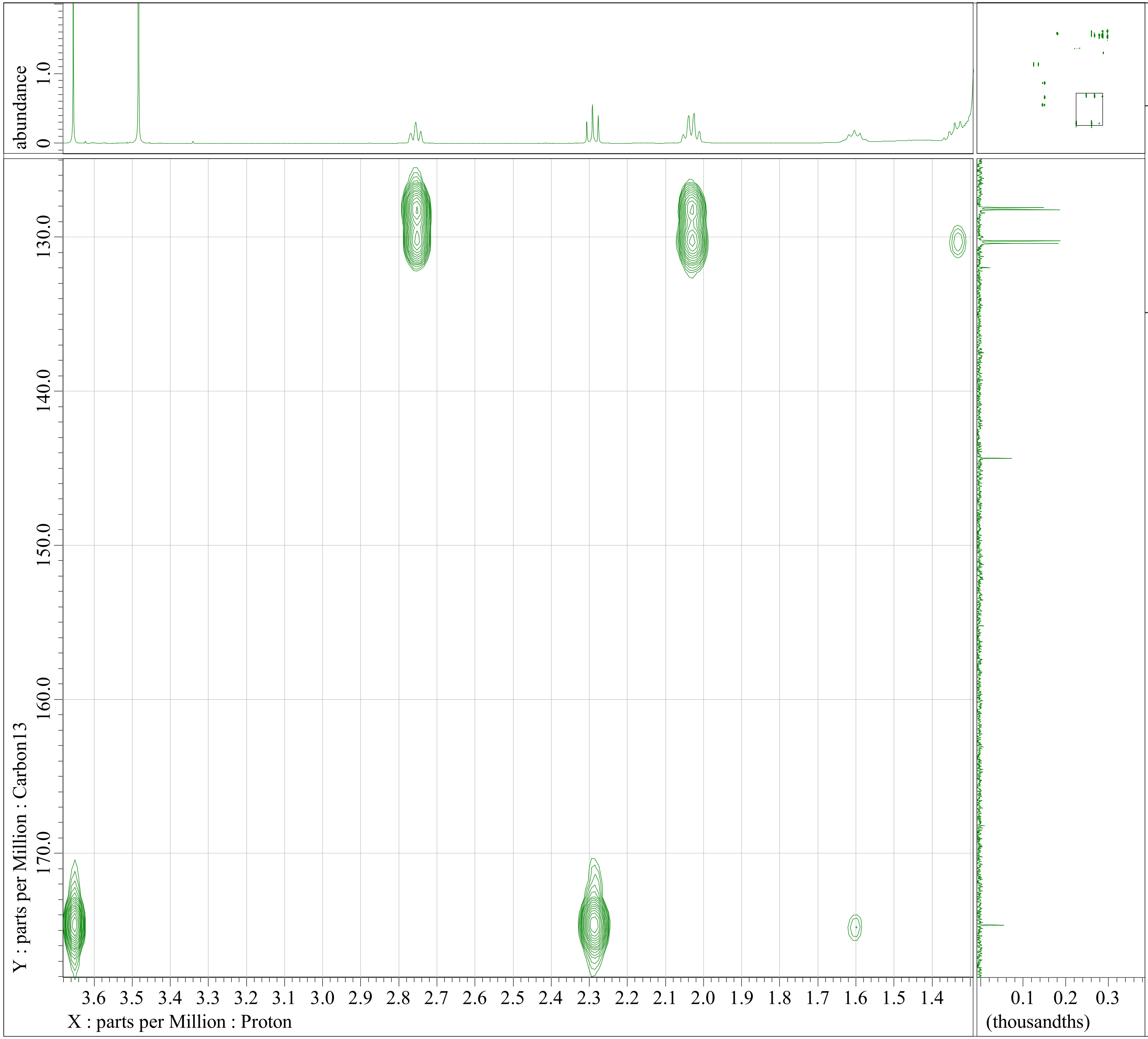

JEOL

```
---- PROCESSING PARAMETERS ----
gauss( 5.0[Hz], 0.0[s] )
sinbell_auto
zerofill( 1 )
fft( 1, TRUE, TRUE )
ppm
[transpose]
sinbell4( -60, 160 )
trapezoid( 0[%], 5[%], 80[%], 100[%] )
zerofill( 2 )
fft( 1, TRUE, TRUE )
ppm
abs
```

```
Filename             = PYR-ML-CH3OH-PYR 2-U_
Author               = delta
Experiment           = hmbc.jxp
Sample_Id            = PYR-ML-CH3OH-PYR 2-U
Solvent              = CHLOROFORM-D
Actual_Start_Time    = 18-JUN-2019 15:15:44
Revision_Time        = 18-JUN-2019 16:26:40

Comment              = gradient enhanced HMB
Data_Format          = 2D REAL REAL
Dim_Size             = 1638, 512
X_Domain             = Proton
Y_Domain             = Carbon13
Dim_Title            = Proton Carbon13
Dim_Units            = [ppm] [ppm]
Dimensions           = X Y
Spectrometer         = JNM-ECZ500R/S1

Field_Strength       = 11.7473579[T] (500[MH
X_Acq_Duration       = 0.21823488[s]
X_Domain             = Proton
X_Freq               = 500.15991521[MHz]
X_Offset             = 5.0[ppm]
X_Points             = 2048
X_Prescans           = 4
X_Resolution         = 4.58221894[Hz]
X_Sweep              = 9.38438438[kHz]
X_Sweep_Clipped      = 7.50750751[kHz]
Y_Domain             = Carbon13
Y_Freq               = 125.76529768[MHz]
Y_Offset             = 100[ppm]
Y_Points             = 256
Y_Prescans           = 0
Y_Resolution         = 122.83805031[Hz]
Y_Sweep              = 31.44654088[kHz]
Tri_Domain           = Proton
Tri_Freq             = 500.15991521[MHz]
Tri_Offset           = 5.0[ppm]
Blanking             = 2[us]
Clipped              = FALSE
Scans                = 8
Total_Scans          = 2048

Relaxation_Delay     = 1.5[s]
Recvr_Gain           = 76
Temp_Get             = 21.7[dC]
X_Acq_Time           = 0.21823488[s]
X_Atn                = 8.9[dB]
```

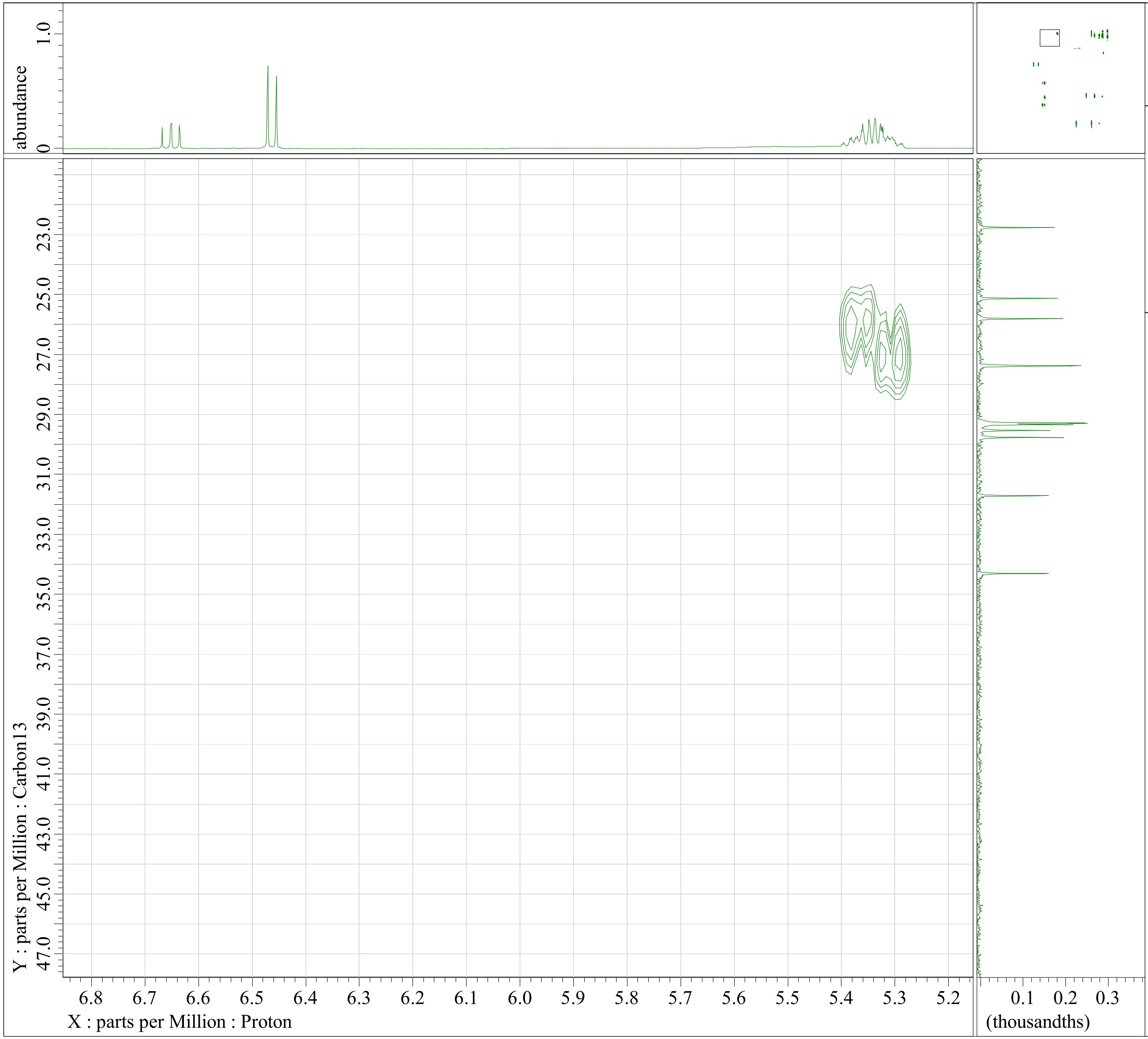

JEOL

```
---- PROCESSING PARAMETERS ----
sexp( 0.2[Hz], 0.0[s] )
trapezoid( 0[%], 0[%], 80[%], 100[%] )
zerofill( 1 )
fft( 1, TRUE, TRUE )
machinephase
ppm
```

```
Filename              = PYR-ML-CH3OH-PYR 2-U_
Author                = delta
Experiment            = hmbc.jxp
Sample_Id             = PYR-ML-CH3OH-PYR 2-U
Solvent               = CHLOROFORM-D
Actual_Start_Time     = 18-JUN-2019 15:15:44
Revision_Time         = 18-JUN-2019 16:26:40

Comment               = gradient enhanced HMB
Data_Format           = 2D REAL REAL
Dim_Size              = 1638, 512
X_Domain              = Proton
Y_Domain              = Carbon13
Dim_Title             = Proton Carbon13
Dim_Units             = [ppm] [ppm]
Dimensions            = X Y
Spectrometer          = JNM-ECZ500R/S1

Field_Strength        = 11.7473579[T] (500[MH
X_Acq_Duration        = 0.21823488[s]
X_Domain              = Proton
X_Freq                = 500.15991521[MHz]
X_Offset              = 5.0[ppm]
X_Points              = 2048
X_Prescans            = 4
X_Resolution          = 4.58221894[Hz]
X_Sweep               = 9.38438438[kHz]
X_Sweep_Clipped       = 7.50750751[kHz]
Y_Domain              = Carbon13
Y_Freq                = 125.76529768[MHz]
Y_Offset              = 100[ppm]
Y_Points              = 256
Y_Prescans            = 0
Y_Resolution          = 122.83805031[Hz]
Y_Sweep               = 31.44654088[kHz]
Tri_Domain            = Proton
Tri_Freq              = 500.15991521[MHz]
Tri_Offset            = 5.0[ppm]
Blanking              = 2[us]
Clipped               = FALSE
Scans                 = 8
Total_Scans           = 2048

Relaxation_Delay      = 1.5[s]
Recvr_Gain            = 76
Temp_Get              = 21.7[dC]
X_Acq_Time            = 0.21823488[s]
X_Atn                 = 8.9[dB]
```

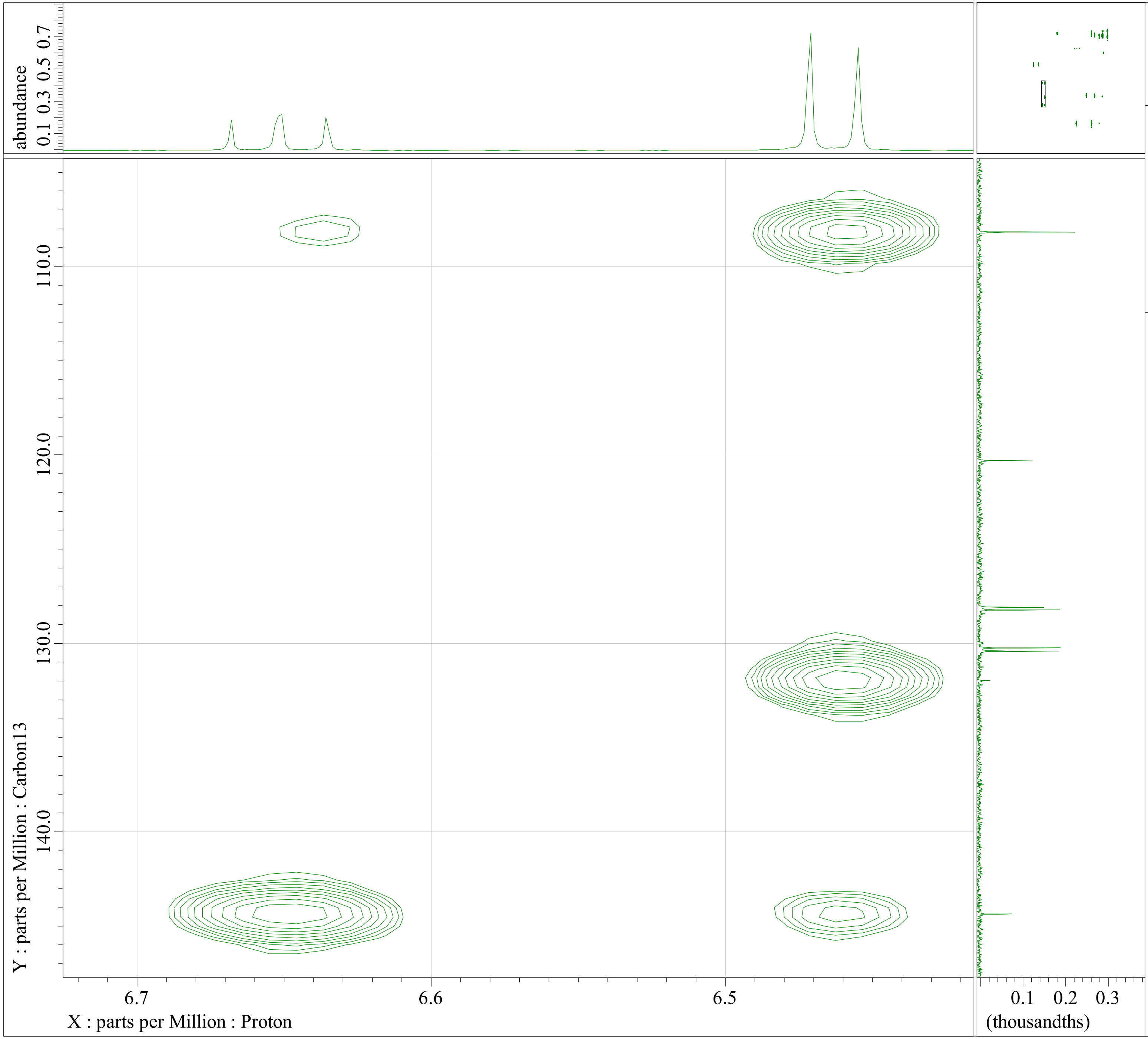

---- PROCESSING PARAMETERS ----
sexp(0.2[Hz], 0.0[s])
trapezoid(0[%], 0[%], 80[%], 100[%])
zerofill(1)
fft(1, TRUE, TRUE)
machinephase
ppm

Filename	= PYR-ML-CH3OH-PYR 2-U_
Author	= delta
Experiment	= hmbc.jxp
Sample_Id	= PYR-ML-CH3OH-PYR 2-U
Solvent	= CHLOROFORM-D
Actual_Start_Time	= 18-JUN-2019 15:15:44
Revision_Time	= 18-JUN-2019 16:26:40
Comment	= gradient enhanced HMB
Data_Format	= 2D REAL REAL
Dim_Size	= 1638, 512
X_Domain	= Proton
Y_Domain	= Carbon13
Dim_Title	= Proton Carbon13
Dim_Units	= [ppm] [ppm]
Dimensions	= X Y
Spectrometer	= JNM-ECZ500R/S1
Field_Strength	= 11.7473579[T] (500[MH
X_Acq_Duration	= 0.21823488[s]
X_Domain	= Proton
X_Freq	= 500.15991521[MHz]
X_Offset	= 5.0[ppm]
X_Points	= 2048
X_Prescans	= 4
X_Resolution	= 4.58221894[Hz]
X_Sweep	= 9.38438438[kHz]
X_Sweep_Clipped	= 7.50750751[kHz]
Y_Domain	= Carbon13
Y_Freq	= 125.76529768[MHz]
Y_Offset	= 100[ppm]
Y_Points	= 256
Y_Prescans	= 0
Y_Resolution	= 122.83805031[Hz]
Y_Sweep	= 31.44654088[kHz]
Tri_Domain	= Proton
Tri_Freq	= 500.15991521[MHz]
Tri_Offset	= 5.0[ppm]
Blanking	= 2[us]
Clipped	= FALSE
Scans	= 8
Total_Scans	= 2048
Relaxation_Delay	= 1.5[s]
Recvr_Gain	= 76
Temp_Get	= 21.7[dC]
X_Acq_Time	= 0.21823488[s]
X_Atn	= 8.9[dB]

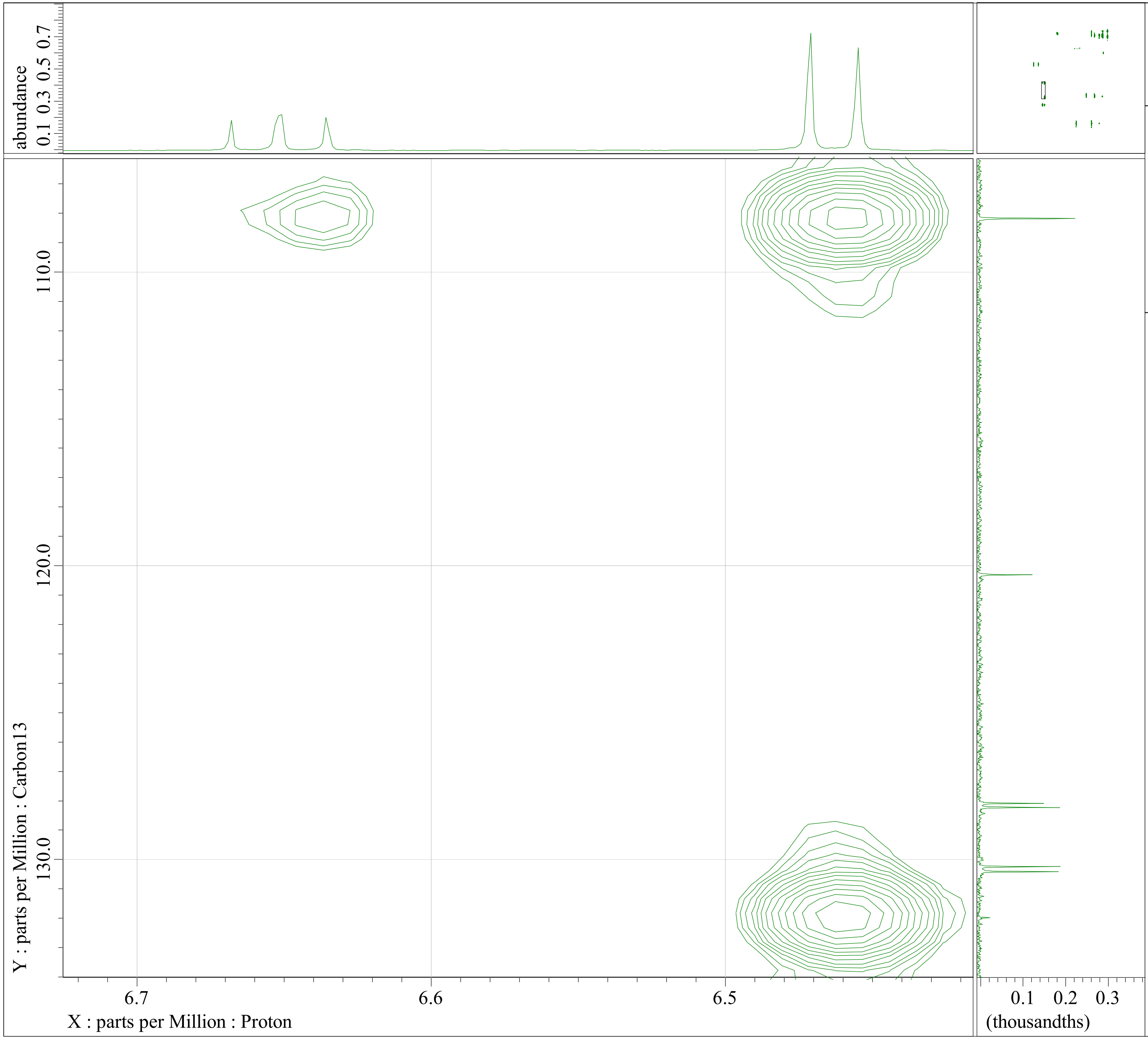


```
---- PROCESSING PARAMETERS ----
gauss( 5.0[Hz], 0.0[s] )
sinbell_auto
zerofill( 1 )
fft( 1, TRUE, TRUE )
ppm
[transpose]
sinbell4( -60, 160 )
trapezoid( 0[%], 5[%], 80[%], 100[%] )
zerofill( 2 )
fft( 1, TRUE, TRUE )
ppm
abs
```

```
Filename              = PYR-ML-CH3OH-PYR 2-U_
Author                = delta
Experiment            = hmbc.jxp
Sample_Id             = PYR-ML-CH3OH-PYR 2-U
Solvent               = CHLOROFORM-D
Actual_Start_Time     = 18-JUN-2019 15:15:44
Revision_Time         = 18-JUN-2019 16:26:40

Comment               = gradient enhanced HMB
Data_Format           = 2D REAL REAL
Dim_Size              = 1638, 512
X_Domain              = Proton
Y_Domain              = Carbon13
Dim_Title             = Proton Carbon13
Dim_Units             = [ppm] [ppm]
Dimensions            = X Y
Spectrometer          = JNM-ECZ500R/S1

Field_Strength        = 11.7473579[T] (500[MH
X_Acq_Duration        = 0.21823488[s]
X_Domain              = Proton
X_Freq                = 500.15991521[MHz]
X_Offset              = 5.0[ppm]
X_Points              = 2048
X_Prescans            = 4
X_Resolution          = 4.58221894[Hz]
X_Sweep               = 9.38438438[kHz]
X_Sweep_Clipped       = 7.50750751[kHz]
Y_Domain              = Carbon13
Y_Freq                = 125.76529768[MHz]
Y_Offset              = 100[ppm]
Y_Points              = 256
Y_Prescans            = 0
Y_Resolution          = 122.83805031[Hz]
Y_Sweep               = 31.44654088[kHz]
Tri_Domain            = Proton
Tri_Freq              = 500.15991521[MHz]
Tri_Offset            = 5.0[ppm]
Blanking              = 2[us]
Clipped               = FALSE
Scans                 = 8
Total_Scans           = 2048

Relaxation_Delay      = 1.5[s]
Recvr_Gain            = 76
Temp_Get              = 21.7[dC]
X_Acq_Time            = 0.21823488[s]
X_Atn                 = 8.9[dB]
```
